# Cross-Site Comparison of Land-Use Decision-Making and Its Consequences across Land Systems with a Generalized Agent-Based Model

**DOI:** 10.1371/journal.pone.0086179

**Published:** 2014-01-29

**Authors:** Nicholas R. Magliocca, Daniel G. Brown, Erle C. Ellis

**Affiliations:** 1 Department of Geography and Environmental Systems, University of Maryland, Baltimore County, Baltimore, Maryland, United States of America; 2 School of Natural Resources and Environment, University of Michigan, Ann Arbor, Michigan, United States of America; 3 The National Socio-Environmental Synthesis Center (SESYNC), Annapolis, Maryland, United States of America; Cinvestav-Merida, Mexico

## Abstract

Local changes in land use result from the decisions and actions of land-users within land systems, which are structured by local and global environmental, economic, political, and cultural contexts. Such cross-scale causation presents a major challenge for developing a general understanding of how local decision-making shapes land-use changes at the global scale. This paper implements a generalized agent-based model (ABM) as a virtual laboratory to explore how global and local processes influence the land-use and livelihood decisions of local land-users, operationalized as settlement-level agents, across the landscapes of six real-world test sites. Test sites were chosen in USA, Laos, and China to capture globally-significant variation in population density, market influence, and environmental conditions, with land systems ranging from swidden to commercial agriculture. Publicly available global data were integrated into the ABM to model cross-scale effects of economic globalization on local land-use decisions. A suite of statistics was developed to assess the accuracy of model-predicted land-use outcomes relative to observed and random (i.e. null model) landscapes. At four of six sites, where environmental and demographic forces were important constraints on land-use choices, modeled land-use outcomes were more similar to those observed across sites than the null model. At the two sites in which market forces significantly influenced land-use and livelihood decisions, the model was a poorer predictor of land-use outcomes than the null model. Model successes and failures in simulating real-world land-use patterns enabled the testing of hypotheses on land-use decision-making and yielded insights on the importance of missing mechanisms. The virtual laboratory approach provides a practical framework for systematic improvement of both theory and predictive skill in land change science based on a continual process of experimentation and model enhancement.

## Introduction

Global environmental change and economic globalization are increasingly coupled with local land-use and livelihood transitions [1]–[3]. Managing the sustainability of such transitions requires an understanding of both the local realities of changing land-use and livelihood patterns, as well as the larger-scale contexts structuring local decision-making [4]–[7]. This relates to a fundamental challenge in land change science (LCS): to produce systematic knowledge of how and under what conditions local land change trajectories are constrained by local land systems, and how responsive these are to changing global forcings [8], [9]. To overcome this challenge, the LCS community has focused on cross-site comparisons and synthesis of case study knowledge in its efforts to reveal commonalities and differences among local land system change patterns and processes and to build land system change theory in general [10]. Yet the ability of LCS to systematically assess the causes and consequences of local land system change at global scale remains hampered by the fragmentation of knowledge on land system change across the local case-study literature. Here, we conduct a cross-site comparison of the adaptive responses of agents to varying local and global economic, environmental, and demographic conditions using a generalized agent-based model (ABM) applied to real world sample landscapes using a virtual laboratory approach. Our goal is to generate and test hypotheses about where and when local contextual complexities are and are not needed to explain local land-use and livelihood patterns globally.

Generating systematic knowledge of the causes and consequences of local land-use and livelihood change (i.e. land system change) globally faces multiple simultaneous challenges. Land system change at the local scale is influenced by a wide array of driving forces often found to be highly dependent on local context, which makes generalizations about the forces that drive change at the global scale difficult [11]–[15]. Mismatches between the resolution of remote sensing data on land cover and the spatial and temporal scales of important social and/or biophysical processes, for example, plague studies of land system change [13], which inherently must account for a diverse range of causal explanations. The best available data representing global scale drivers of land system change, such as market influence [16], are too coarse to represent the market-oriented land-use decisions of individual land users. More fundamentally, the impossibility of widespread experimental manipulations of land systems, the multitude of forces influencing land system change, and the complexity of their interactions represent major obstacles to connecting land-use changes to their causes at and between local, regional, and global scales [13]. Consequently, substantial uncertainty exists surrounding the mechanisms through which global and regional drivers influence local land-use decisions, and how such relationships and their effects may or may not vary by location.

Cross-site comparison and synthesis methods, such as meta-analysis, can identify common patterns across empirical case studies, and have thus far been used in LCS through a mix of loosely structured meta-study techniques. Meta-studies of land system change vary from fully quantitative statistical analyses (e.g. [11], [17]) to qualitative coding methods such as qualitative comparative analysis (e.g. [18]). Regardless of the synthesis method, the ability to make systematic comparisons is ultimately limited by the consistency of the methods, documentation, and various theoretical lenses used to conduct case studies of land system change. Cases studies are performed across different spatial and temporal scales and from the perspectives of many varied disciplines [10]. No standard case-study methodology exists in LCS, which leaves the interpretation and coding of drivers of land system change open to the meta-analyst [19]. Even when such synthesis methods successfully identify common patterns across empirical case studies, they cannot provide mechanistic explanations of how such empirical patterns emerge from underlying processes, and thus lack the means to form and test hypotheses of how such systems will respond locally to changing large-scale forces.

Rindfuss and colleagues [20] propose that simulation models of land-use change, and agent-based models (ABMs) in particular because of their representation of human decision-making processes, provide a more formal means of comparison. In response, Parker and colleagues [10] made the first attempt at a systematic comparison of ABMs of land-use change in frontier regions. The comparison was based on how each model addressed agent-parcel relationships, non-spatial social networks, land suitability, multiple agents, land transfer mechanisms, and institutional drivers. However, just as meta-studies are constrained by lack of standardization across case studies, these comparisons revealed inconsistencies in how the same processes/structures were represented across models. Although this comparison was constrained by the limited scope and scale representations of the input models, the potential of ABMs as a viable means of comparison across sites was demonstrated.

Using ABMs as a means of comparison brings additional challenges. The validity of any ABM is dependent on the specification of agents' decision-making rules and interactions [21]. In an effort to make ABMs more realistic, agents' decision-making rules might be parameterized to conform with individual-level empirical data, such as characteristics associated with agent typologies (e.g. [22]). This imposes significant data demands, which reduces the model's domain of applicability because the modeler is tempted to calibrate, and possibly overfit, it to the observed patterns in a particular system [6], [23], [24]. Furthermore, the empirical data and process knowledge needed to formalize linkages between agents' land-use and livelihood decision-making and global-level forces, and to systematically compare land system change trajectories across sites, is lacking.

A major challenge for model-based comparison, then, is to find the proper balance between the number and types of interactions represented and the generality of their representation. Building on the concept of pattern-oriented modeling (POM) [25] in a virtual laboratory setting [8], [26] for designing, parameterizing, and testing multi-scale ABMs with limited data, we conducted comparative experiments across multiple sites and diverse land systems. The virtual laboratory is applied to six test sites to illustrate this approach and analyze the causes of landscape outcomes across a wide range of environmental and social conditions that are impossible to control and experiment with in the field. We test an initial hypothesis that a minimal set of local and global demographic, environmental, and market conditions is sufficient to structure agent decisions such that stable strategies emerge that reproduce observed land-use and livelihood patterns across sites. The overall goal is to reproduce the land-use and livelihood patterns observed at one point in time for each of the sites, and by doing so provide insight into the decision-making processes that produced those patterns. Model performance then guides the formation of additional hypotheses of the relative importance and predictability of local versus global factors in determining land-use and livelihood patterns for future testing across sites. The next section describes the selection and characteristics of the set of test sites investigated. This is followed by an overview of the procedures used to parameterize the general model for each test site, and the experimental and statistical frameworks used to compare model results across sites. The following section discusses the sources of and insights from model errors, and which factors were important for shaping land-use patterns within and across sites. We conclude with a discussion of the potential for this virtual laboratory approach to advance cross-site comparison and theory-building efforts in LCS.

## Materials and Methods

### 2.1. Site selection

**Figure 1:**
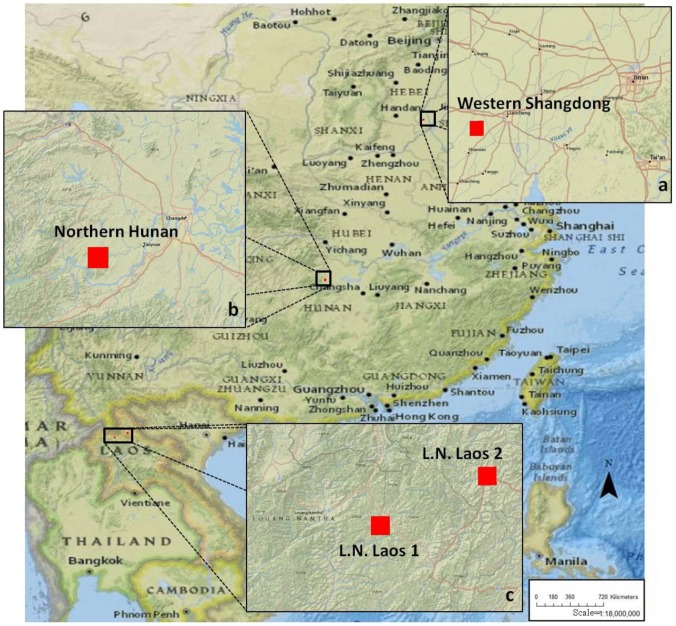
Locations of eastern Asian sites: two in China (western Shandong Province, China (a) and Northern Hunan Province, China (b)), two in Luoang Namtha, Laos (c).

**Figure 2:**
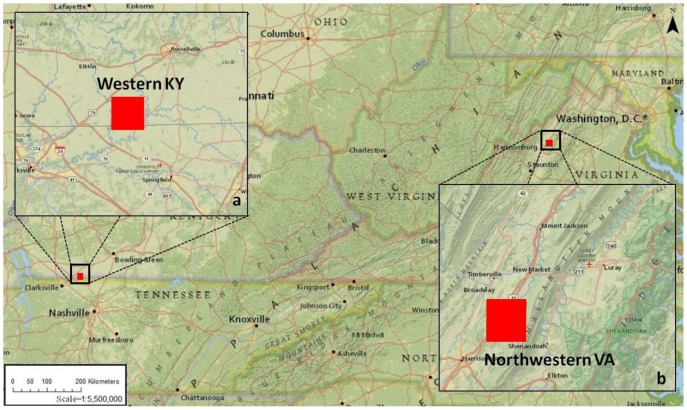
Location of U.S. sites: one in southwestern Kentucky (a), and one in northwestern Virginia (b).

Six test sites were selected across the approximate range of variation observed in a set of global environmental [27]–[29], population density [30], and market influence index [16] variables (Table 1). Global market influence is sampled directly from Verburg et al. [16] as a combination of market access and a market influence index (based on travel time to large cities and purchasing power parity, respectively; see [16] for description), and is normalized to values between 0 to 1.Sites were chosen such that two sites occupied each variable class, and sites within the same region/country occupied different slope classes. Sample sites included two in China (western Shandong Province, China (1a) and northern Hunan Province, China (1b)), two in Luoang Namtha, Laos (1c), one in southwestern Kentucky, USA (2a), and one in northwestern Virginia, USA (2b) (Figures 1 and 2; Table 2). Land-cover data were obtained from classified Landsat images from 2001 NLCD [31] for U.S. sites, Heinimann et al. [32] for the Laos sites, and Ellis et al. [33] for the China sites.

**Table 1:** Selection criteria for test sites.

Selection Criteria	Class Values	Number of Sites
Population Density Classes (people km^−2^)	< = 16	2 sites in each class across sample set
	>16 & < = 100	
	>100	
Market Influence Classes (index value)	< = 0.35	2 sites in each class across sample set
	>0.35 & < = 0.65	
	>0.65	
Slope Classes (%)	< = 8	Different slope classes for each site per pair of sites in the same region
	>8 & < = 16	
	>16	

**Table 2:** Summary statistics for each test site.

Site Name		W. Shangdong	N. Hunan	W. KY	N.W. VA	L.N. Laos 1	L.N. Laos 2
Latitude		36°26′4″N	28°54′27″N	36°43′14″N	38°35′4″N	20°55′21″N	21°10′8″N
Longitude		115°40′35″E	111°12′35″E	87°5′12″W	78°47′46″W	101°37′7″E	102°12′37″E
Population Density (ppl km^−2^)	Min	73.8	1.2	0	0	1.23	0
	Mean	**512.3** a	**124.2**	**4.6**	**22.4**	**36.7**	**12.9**
	Max	4,113.0	2,777.6	161.4	314.0	2,160.7	180.0
Shannon's Evenness Index for Population Distrbution. (Equal Distr. = 1.0)		0.9570	0.8781	0.7753	0.8993	0.8018	0.8499
Market Influence	Min	0.60	0.51	0.83	0.84	0.02	0.02
	Mean	**0.63**	**0.59**	**0.86**	**0.87**	**0.02**	**0.02**
	Max	0.71	0.67	0.88	0.88	0.02	0.06
Market Access	Min	0.14	0.06	0.40	0.44	0	0
	Mean	**0.29**	**0.17**	**0.59**	**0.62**	**0**	**0**
	Max	0.71	0.41	0.73	0.74	0	0
Slope (%)	Min	0	0	0	0	0	0.50
	Med.	**9.8**	**11.3**	**7.2**	**11.7**	**17.5**	**40.2**
	Max	70.0	45.9	79.8	85.3	105.1	123.3
Land Suitability Classes (%)	1	**47.8**	37.9	**57.8**	28.7	28.5	4.1
	2	39.1	**41.2**	34.0	**38.7**	19.1	5.0
	3	12.4	19.1	7.6	26.4	**29.9**	18.5
	4	0.7	1.8	0.6	6.2	22.5	**72.4**

a Bolded values are the mean values and dominant land suitability class (class 1 = most suitable for agriculture; class 4 = least suitable for agriculture). Coordinates are provided for the top-left corner of the test site bounding boxes.

### 2.2. Site Descriptions

The first site in China was located in the prime agricultural areas of the North China Plain near the town of Liaocheng in Shandong Province (Fig. 1a). The site is characterized by nearly uniformly distributed dense populations (Table 2) concentrated in small villages around which land cover was dominated by intensive cultivation. The second site in China was located near the town of Taoyuan in the hilly regions of northern Hunan Province in south central China (Fig. 1b). The site is characterized by fairly high population density dispersed within and along the edges of two main valleys. Intensive cultivation of rice is present around settlement areas, while extensive cultivation occupies areas with moderate slopes on the edges of valleys. The first U.S. site was located in prime agricultural areas along the border of Kentucky and Tennessee near Russellville, KY (Fig. 2a). The site was characterized by abundant prime agricultural land and dominated by the commercial cultivation of corn and soybeans. The second U.S. site was located in the Massanutten Mountains near Harrisionburg, VA (Fig. 2b). Due to the short growing season and hilly terrain, land-use/cover is dominated by pasture and forest cover. The terrain is fairly hilly with median slopes of 11.7 percent (Table 2). Population density is low and concentrated around a few small towns. Market influence and access are both high because of the several large roads that intersecting the site. The final two sites were in northern Laos. The first was a combined rice and swidden cultivation system in the Luoang Namtha region of northern Laos (Fig. 1c). The site was characterized by dispersed pockets of high population density (Table 2) in proximity to intensive rice cultivation, while the rest of the landscape was sparsely populated and forested or cultivated with low labor inputs. The second site in Laos was located in a mainly swidden cultivation system in the Luoang Namtha region of northern Laos (Fig. 1c). The site was characterized by very hilly terrain (median slope of 40.2 percent) with patches of extensive cultivation dispersed across the landscape. Detailed site-specific descriptions and results can be found in the Section S2 in Supplementary Information S1.

### 2.3. Global data inputs

The global context of each site was represented with several global datasets that were used as input to the ABM and that ensured cross-site comparability. Publicly available global datasets (Table 3) were re-sampled for each site in ArcGIS 10.0 using zonal statistics to the spatial resolution of 100 m in the local WGS 1984 UTM projection. Potential agricultural yields are based on a global dataset of observed yields [34] and then modified according to local terrain and precipitation constraints on agricultural production. High resolution (∼30 m) topographic data from the ASTER Global DEM [28] is used to determine slope. Slope is a proxy for suitability of soil for agriculture with reductions in potential agricultural yields based on Global Agro-Ecological Zones (GAEZ) slope constraint classes [28]. Precipitation levels during the growing season [29] may impose additional reductions in potential agricultural yields. Combined slope and precipitation constraints were used to create an agricultural suitability layer used to modify the potential productivities of land uses. Global market access is re-sampled directly from [16] and the market influence index is normalized to values between 0 and 1.

**Table 3:** Global data inputs used to parameterize the model environment.

Input Data	Description	Native Resolution	Source
Population Density	LandScan 2000 population density model	30 arc-second	[31]
Market Access and Influence	Based on travel time to large cities and purchasing power parity, respectively	30 arc-second	[16]
Potential Agricultural Yields	Climatic potential wheat yields	5 arc-minute	[35]
Slope	Percent slope calculated from DEM	30 meter	[28]
Land Suitability	Percent reduction in potential agricultural yields due to slope and precipitation constraints	30 arc-second	[29]
Precipitation Constraints	Average rainfall during growing season	30 arc-second	[30]
Land Use/Cover	Classified LandSat images	30 meter	[32]–[34]

## Model Analysis

The generalized ABM of land-use and livelihood decision-making developed in Magliocca et al. [26] was applied to the six landscapes described above to assess how global market influence interacts with local environmental and demographic conditions to affect local land-use patterns. Landscape outcomes were modeled as a result of the decisions of agents representing aggregates of households, in annual increments over a twenty-year period (with the first ten as model spin-up). Agents' behavioral rules were derived from smallholder household economic theories [35], [36], and involved allocation of labor to on-farm subsistence and market production, as well as off-farm wage labor based on the expected payoff from each of these activities and heterogeneous risk preferences. Agents endogenously learned and adapted to the utility-maximizing land-use and livelihood strategies for their locations within the given landscapes. Agents land-use and livelihood decisions were exogenously structured by local population densities and environment conditions, which influenced agricultural productivity and land availability per capita; as well as global market forces, which influenced crop and agricultural input prices, wage rates, and transportation costs. Agro-ecological dynamics emerged from agent-environment interactions, which in turn provided feedbacks to agents' decisions and resulted in the evolution of stable land-use and livelihood strategies by the end of the model simulations. Detailed model specifications, descriptions of agents' decision heuristics and attributes, and psuedocode are provided in Magliocca et al. [26].

Since little is known about how economic globalization explicitly interacts with local environmental and demographic conditions to affect decisions, this paper builds on the approach of using POM within a virtual laboratory [8] to design, parameterize, and test multi-scale ABMs of land-use change. This approach is used to test alternative representations of local land-use decision making through a set of cost and price functions, which are parameterized using a genetic algorithm subject to performance criteria drawn from the empirical livelihoods and development literatures (see [8] and [26] for details). In the initial implementation of this model [26], global market influence was shown to be the main driver of livelihood strategies, which interacted with local environmental conditions and population density to structure agents' choices of the most efficient land-uses.

### 3.1. Parameterizing and Modeling Test Sites

Land-use outcomes are modeled as the result of livelihood and land-use decisions of agents at annual increments over a twenty-year period (after ten periods for model spin-up). For each test site, a raster landscape of 100 by 100 hectare-sized cells was generated using ArcGIS 10.0. One hundred agents were each assigned a ‘settlement area’ of 10 by 10 cells (1 km^2^), over which their land-use choices were made. This simplification into “settlement agents” was made to easily manipulate population density and land per capita settings, and test the effects of land allocation processes across sites.

High resolution land-cover data (∼30 m) were obtained from a variety of sources specific to each site (Table 3; also see Section S1 in Supplementary Information S1 for site-specific details). Seven different land use/cover categories were represented in the model: three productive uses (intensive agriculture, extensive agriculture, and pasture for grazing livestock) and four non-productive uses (forest, fallow, dwellings/urban, and non-use [water, barren/rock]). Productive land uses were defined by functional group, rather than particular types (e.g. ‘intensive’ and ‘extensive’ versus irrigated rice or shifting cultivation based on cassava), which vary in their potential productivity, degradation/regeneration rates, and labor and input costs [26]. Land-cover categories from the data were re-classified to align with those represented in the model as closely as possible by combining agricultural land cover classes into functional groups. The model was initialized with non-productive uses in the same locations as in the real landscape, but all other cells were set to the lowest labor input agricultural use (i.e. extensive cultivation).

### 3.2. Model Experiments

Different representations of market influence and population density were experimentally manipulated to test the conditions under which model-derived land-use patterns best matched those observed for each site. Population and market were represented as 1) spatially variable and equal to the values for the real landscape, 2) uniform across the landscape and equal to the mean value of the real landscape, or 3) uniform and plus or minus 0.1 and 0.2 different from the observed mean market influence and plus or minus 10 and 20 percent away from the observed population density values (model settings: 1+5×5 = 26). For each site, the model was run 60 times for each possible population and market combination (n = 1,560) in order to find unique combinations of cost and price function parameters generated by the genetic algorithm that met model performance criteria according to the POM approach (see [8] and [27] for details). The results from these model settings were contrasted with a random null model, in which each land-use/cover category had equal probability of occurring in each cell.

Population levels did not change during the simulation, and crop prices and yields were held constant within a given model run to allow agents to learn stable land-use and livelihood strategies under alternative conditions. Variability in crop prices and yields, for example, are certainly important influences of land-use choices and livelihood strategies. However, exploring responses to such variability is beyond the scope of this paper, because the focus here lies in generating the land-use and livelihood strategies that are best suited to alternative conditions across sites in an attempt to reproduce observed patterns.

This experimental design explored the sensitivity of modeled land-use outcomes to variations in the ways model input information was represented (spatially variable vs. uniform in various ways). If modeled processes accurately represented those operating at the test sites, then one would expect model outcome accuracy to improve as model inputs more closely resembled those observed. Conversely, if model outcome accuracy declined as model inputs more closely resembled those observed in reality, then one or more modeled processes may have been incorrectly represented. Testing the model with alternative inputs allowed for differentiation between accurate model outcomes generated by realistic process representation versus model artifacts. For example, alternative model settings tested the effects of imposing a ‘settlement area’ for each agent, which assumed that production activities were concentrated within one square kilometer of population centers. If this was the case, then the spatially explicit representation was expected to produce land-use patterns that best matched those observed. However, if production activities expanded beyond the imposed ‘settlement area’, then land-use intensity would differ from that expected given the observed population density. Finally, the alternative population and market setting were use to explore the stability of aggregate land-use outcomes resulting from agents' cumulative adaptive decisions in response to conditions beyond those observed in the current landscapes.

### 3.3. Statistical Analysis

Several descriptive statistics were calculated for each site, measuring the differences in distributions and rankings of each land-use/cover category between the real and modeled landscapes. Only intensive agriculture, extensive agriculture, pasture, forest, and fallow land-use/cover classes were considered in statistical analyses. Dwellings, barren/rock, and water were excluded because they were initialized in the modeled landscape exactly as in the real landscape and held constant throughout the simulation. Modeled land-use outcomes, including the null landscape, were compared to the real landscape to measure the added explanatory power of the process-based model. To compare aggregate landscape counts of each land-use/cover category, a simple multi-dimensional distance measure, *D*, is used.
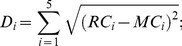
(1)where *RC_i_* and *MC_i_* are counts of each land-use/cover category, *i*, in the real and modeled landscapes, respectively. A more detailed assessment of model error for each site was performed by disaggregating Eq. 1 into land-use/cover counts within each land suitability, which is provided in Section S1 in Supplementary Information S1.

In addition, agreement between the rankings of land-use/cover category counts for real and modeled landscapes was determined using Spearman's ranked correlation. If a strong positive correlation exists between the rankings, then the model matches the ranked abundances of categories observed in the real landscape.

Finally, the degree to which land-use decisions were affected more by market influence versus population pressure was measured by comparing the relative value of agricultural products per land and labor unit inputs (i.e. ‘returns to labor’) in response to market and population settings. Returns to labor were calculated from each site's observed yields, which reflect the intensity of production subject to land suitability, and the value of agricultural production, which reflects market influence settings. For simplicity, an aggregate return to labor is presented for an entire site. However, within sites individual agent's returns to labor varied subject to heterogeneous preferences for and perceptions of risk and return in market opportunities.

These measures were intended to provide descriptions of model performance within and across sample sites and not to test statistical hypotheses of independence or similarity between real and modeled landscape metrics. At this stage of hypothesis generation, statistical significance is not yet relevant, as many important mechanisms known to influence patterns of LUCC are not included in the model design. Instead, these statistical measures quantify the particular ways in which modeled landscapes deviate from the test site landscapes, which provides insight into potential mechanisms to be included in future model experiments.

## Results

**Figure 3:**
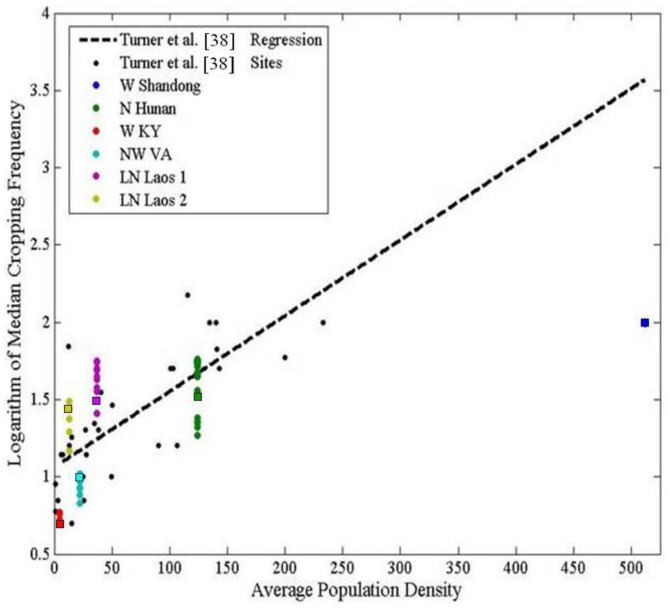
Modeled relationships between cropping frequency and population density were consistent with predictions from Turner et al. [38] across test sites. Multiple outcomes (i.e., points) at each site reflect the mean land-use result for each combination of population and market settings tested. Square data points represent model results from spatially variable model settings.

To verify that agents' assumed decision-making models responded to changing economic, environmental, and demographic influences in realistic ways, the relationship between cropping frequency and population density produced across model runs was compared with that posited by Boserup [37] and empirically tested by Turner et al. [38]. Model outcomes were generally consistent with the predictions of Turner et al. [38]; modeled cropping frequencies increased with increasing average population densities observed across test sites (Fig. 3). However, in the W. Shandong and W. Kentucky sites, cropping frequencies consistent with the general trend of population-driven intensification resulted because the model produced different production activities than those present in the real land systems. Results are discussed in light of these discrepancies in the following section.

### 4.1 Source of model error

**Figure 4:**
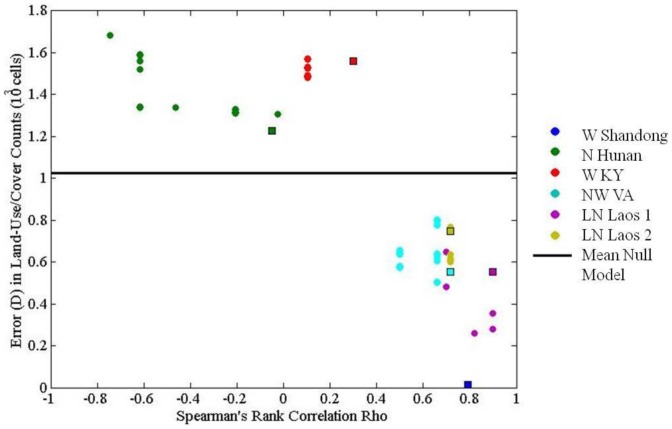
Model error measured as correlation among rankings of and distance (D) between real and mean modeled land-use/cover category counts across sites and compared to the null model. Square data points represent model results from spatially variable model settings.

**Figure 5:**
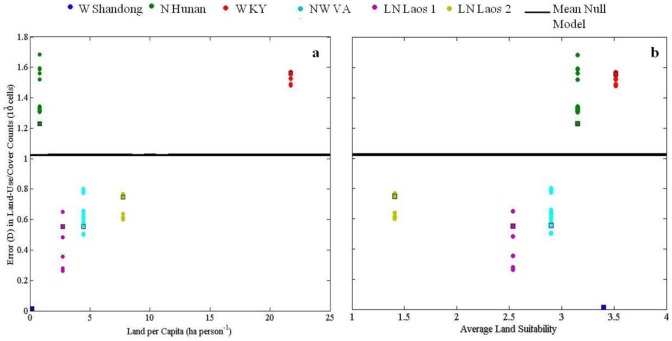
Model performance was better than the null model (black line) for sites in which the imposed ‘settlement area’ approximated the actual land per capita (a) and spatial configurations of land-users' production and consumption activities (i.e. W. Shandong), and/or constraints on land suitability for agriculture were present (b; 4 = no constraints; 1 = severe constraints).

The ABM produced land-use outcomes more consistent with the real landscape than the null model in all but the W. Kentucky and N. Hunan sites (Fig. 4). Results for each site are presented in detail in Supplementary Information S1. Generally, the model performed better than the null landscape for sites that were limited by land suitability (i.e. L.N. Laos 1 and 2, N.W. VA), and/or where the imposed ‘settlement area’ approximated the actual spatial configurations of land-users' production and consumption activities (i.e. W. Shandong) (Fig. 5). Across all sites, spatially variable model settings improved the accuracy of model land-use outcomes, producing rankings of land-use outcomes more similar to the real landscapes and generally less overall error than with spatially uniform model settings.

**Figure 6:**
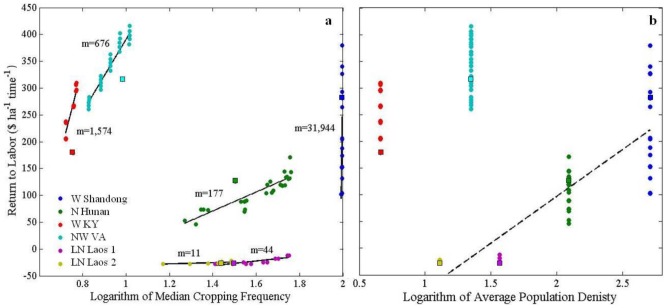
Variations in aggregate returns to labor across sites in relation to median cropping frequency (a) and population density (b) illustrate the relative roles of population pressure and market influence in land-use decisions. The trend line presented with population density (b) serves only to group sites and does not indicate a derived statistical relationship.

Model errors introduced by the ‘settlement area’ simplification were exacerbated when market influence was high. The model performed worse than the null model for land systems in which market influence was an equally strong or stronger driver of land-use decisions than population pressure or environmental constraints (Fig. 6). In Figure 6a, steeper slopes across experimental settings within a given site illustrated the relatively larger influence of market forces over population density on land-use decisions. Flatter slopes indicated intensification decisions driven by population pressure rather than market influence. Figure 6b demonstrates that returns to labor (i.e. value of production per unit of labor) in all Asian sites responded approximately linearly with increasing population density, whereas high returns to labor in the U.S. sites were obtained independent of population pressure.

**Figure 7:**
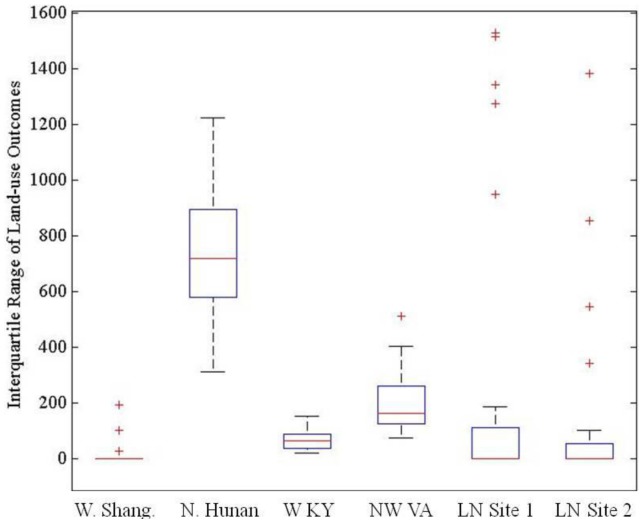
Variations in the values of model inputs (average population density, market influence, and land suitability) and outputs (median cropping frequency, return to labor, and average agricultural yields) standardized to normal scores.

The six test sites differed widely in population density, environmental constraints, and market influence (Table 2). Although these forces were influential in all sites, the relative importance of each in shaping land-use decision-making and outcomes varied across land systems (Fig. 7). Low land suitability significantly constrained land-use outcomes in the Laotian sites, as returns to labor and agricultural yield remained low. In the northern Hunan site, fewer land suitability constraints facilitated intensive cultivation and higher yields. In contrast, land suitability was not a dominant influence on land-use decisions in the remaining sites with more favorable suitability conditions.

Population density was an important structuring process at all Asian sites. Cropping frequencies, agricultural yields, and returns to labor all increased concurrently with population density. When population density and market influence were low, as in the Laotian sites, land-use choices were constrained by labor limitations. Higher population densities in the Chinese sites led to intensified land-use and higher yields. Conversely, population density was not an important driver of land-use decisions for the U.S. sites, as high agricultural yields and returns to labor were obtained at low population densities.

Market influence was an important structuring process in the China and U.S. sites. A large discrepancy between agricultural yields and returns to labor in comparison to the Laotian sites, for which market influence was not a structuring process, was evident. Market influence was an equally important force shaping land-use decisions with environmental constraints and population density for the N. Hunan and W. Shandong sites, respectively. In contrast, market influence was the dominant structuring process on land-use decisions for the U.S. sites as evident by agricultural yields and returns to labor independent of population density and land suitability constraints.

**Figure 8:**
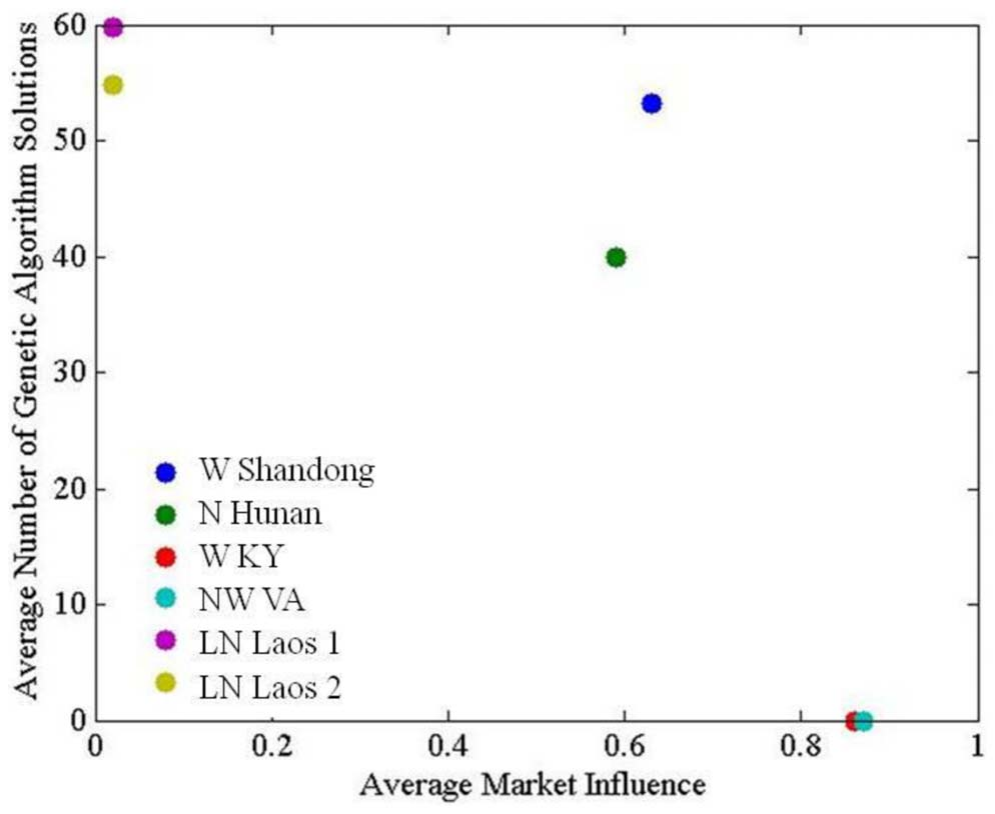
Variations in errors (number of incorrectly modeled cells) in land-use outcomes within each site observed across experimental population density and market influence settings.

The range of experimental settings implemented also enabled exploration of the stability of agents' land-use and livelihood decision-making across settings that varied the level and degree of uniformity in population density and market influence (n = 1,560) for each site. Variability in modeled land-use outcomes, measured as the interquartile range (IQR) of model errors among model runs for each site, illustrated the stability of land-use decisions across experimental population density and market influence settings (Fig. 8). The northern Hunan site showed the largest average variation among model runs, whereas the Laotian sites displayed the largest overall IQR. Detailed analyses of variability across model runs for each site are presented in Supplementary Information S1.

### 4.2 Applicability of ABM v0.1

**Figure 9:**
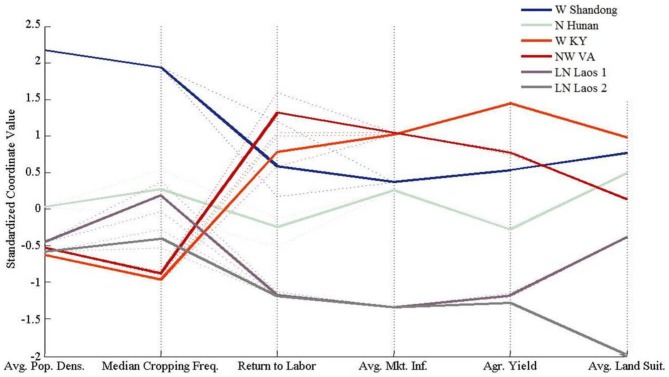
Declining applicability of the underlying smallholder decision-making framework with increasing market influence, measured as the average number of successful genetic algorithm solutions across model runs.

The applicability of the current agent decision-making framework may be limited to land systems in which land-use decisions are structured primarily by population pressure and/or environmental constraints (Figures 6b and 7). This was reflected by the declining success of the genetic algorithm in finding ‘successful’ solutions as market influence increased (Fig. 9). A solution was successful when a parameter set being tested simultaneously reproduced three model performance criteria based on smallholder behavior patterns from the empirical livelihoods and development literatures [8], [26]. Successful solutions were found in at least half of model runs below a market influence of 0.7. Above that point, the genetic algorithm consistently failed to find parameter sets that met all three model performance criteria, as land-use decisions favored profit-maximization rather than labor- and risk-minimization.

## Discussion

Cross-site comparisons of model outcomes confirmed the varying influences of population pressure, environmental constraints, and market influence on land-use decisions across different land systems. Across all sites, the model's performance improved as representations of the level and degree of uniformity in population density and market influence approached those of the real landscape. Combined with favorable comparisons to null model outcomes for four of the six sites, this suggests that the current model has some explanatory power - even with its generalized form. Furthermore, model results illustrated that the applicability of the decision-making framework in our ABM was limited to land systems in which land-use decisions were structured primarily by population pressure and/or environmental constraints. Model settings that increased market influence above those observed in the real landscape led to increased variability in land-use outcomes. Model outcomes produced from these settings suggested that increased market influence may lead to greater dependence on market-oriented livelihood activities, which has the potential to change the composition of the landscape significantly.

Model outcomes also demonstrated how the misrepresentation or exclusion of important processes affected land-use extent and intensity within and across sites. This leads to hypotheses about which land systems require or do not require the addition of context-dependent processes to improve model realism. More importantly, this virtual laboratory approach provides a means for cross-site comparisons of how and under what conditions driving forces of land system change might differ from a generalized model, which leads to testable hypotheses of the global patterns of the relative strength of local versus global context in shaping local land-use and livelihood outcomes. Such questions are currently a focus of efforts to develop spatially explicit, multi-level, and integrated human-natural system global assessment models [39].

### 5.1. Linking Model Errors to Process Representation

The lack of realistic land allocation mechanisms introduced model errors for sites in which 1) land-users' production and consumption activities were disconnected in space, and/or 2) market influence was the primary driver of land-use choices. The imposition of a ‘settlement area’ tended to underestimate the spatial extent of land use and the land per capita ratio. The W. Shandong site was the exception that demonstrated the importance of accurately representing land allocation. The model successfully matched land-use/cover outcomes for this site (Figs. S1 and S2), because the one square kilometer ‘settlement area’ per agent approximated the spatial configuration of villages and resulting average land per capita present in the real system. Thus, population pressure on land-use decisions was accurately modeled, and the intensity and extent of land-use was consistent with what was observed in the test site.

To varying degrees, the ‘settlement area’ simplification distorted land per capita ratios in all other sites. Generally, active use of land was likely more extensive and diffuse than was modeled, and thus model agents responded to population pressures and land supply limitations to a greater degree than real agents. For instance, experimental model settings in which population density was applied evenly over the landscape (and thus more diffusely) produced more realistic land-use outcomes than when the real spatial population distributions were used. For the U.S. sites, which were characterized by high market influence and low population density (Figs. S5, S6, S7, S8), the ‘settlement area’ failed to capture the spatial separation between consumptive and productive land uses characteristic of fully mechanized cultivation systems. Similarly, land tenure likely extends beyond the modeled ‘settlement area’ in the Laotian sites due to the prevalence of low-intensity, extensive land uses (Figs. S9, S10, S11, S12). In the northern Hunan site, the elongated valley-ridge configuration of the landscape produces individual land holdings that are fragmented and distributed both within and outside of the ‘settlement area’ (Figs. S3 and S4). Consequently, modeled land pressure was artificially high in some places, and intensive cultivation was better approximated in model versions with population density settings that were spatially explicit or uniform and slightly less than or equal to the observed mean. The ‘settlement area’ misrepresented land per capita across sites in slightly different ways in different sites, yet intensive cultivation was consistently over-estimated, revealing a common role of land allocation processes.

Model errors provided insights into several other important processes that are currently not represented, yet act in similar ways across land systems to link land-use decisions to landscape outcomes. One of the simplest mechanisms of agricultural intensification in response to population and/or market forces that was omitted and led to errors in modeled agricultural intensity is multi-cropping. Currently, the model only represents up to single cropping without fallow. This limitation was most evident in the W. Shandong site, where modeled cropping frequencies were lower than observed (Fig. 3).

Model errors due to market-driven land-use decisions were more complex, stemming from inadequate representation of mechanisms through which agents respond to market forces. The northwestern Virginia and northern Hunan sites were both moderately limited by land suitability, and in both cases agents responded to market forces by expanding intensive cultivation and/or pasture production. According to the labor- and risk-minimizing decision rationale of the model, pasture was a favorable land-use for market production in these sites because it required relatively low labor/capital inputs, had high returns to labor, and could be produced on marginal land. This logic produced realistic land-use outcomes in northwestern Virginia although the extent of the market response was underestimated due to the ‘settlement area’ simplification.

Because no pasture was present in the real landscape, the expansion of market-oriented pasture production in the model led to large model errors for the northern Hunan site. Two explanations for this discrepancy are possible and not mutually exclusive. First, high population densities favored rice production as the dominant subsistence crop on prime agricultural land, which the model predicted well. The remaining land was less suitable for intensive agriculture, and the model-predicted pasture to be the next best land-use choice for these locations. In reality, low-input cash crops and forestry products, such as tea, bamboo, and/or fir trees, were chosen instead (unpublished data). Cash crops are currently not represented in the model, but results indicate that they strongly influence land-use choices in this location and should be included in future model experiments. A second plausible explanation for model failures is the influence of local land allocation. Historically, land was allocated to individual households evenly based on productivity [40]. Individual land holdings today reflect this legacy, as households manage many small, fragmented areas across the land suitability gradient, and the small plots do not support large grazing pastures [40]. Based on this historical context and the model results, land allocation is clearly an important influence on the types of land uses chosen, and thus should be included in a more detailed model of this location.

Finally, when agents responded to market influence by expanding intensive cultivation, the model could not match the scale of expansion. In reality, agricultural production decisions in such cases are likely oriented towards profit-maximization rather than labor- and risk-minimization through capital for labor substitution. Fully mechanized agriculture was not represented in the model, and thus modeled agricultural intensity for the western Kentucky site was erroneously low. More broadly, expansion of market-oriented land-uses was the main source of variation among model runs within and across sites. As market influence increased, larger variations in potential land-use outcomes emerged (Fig. 8), and the genetic algorithm increasingly failed to find successful solutions (Fig. 9). This illustrated the need to introduce agent decision models with primarily market-oriented objective functions and production rationales capable of making the transition from a labor-driven to capital-driven commercial production system. Combined with the inclusion of common mechanisms through which land-users respond to market forces, such decision-making models will improve the model's ability to represent market-driven land-use changes.

Cross-site comparisons of model performance revealed the relative importance of land suitability constraints, population pressure, and market influence in leading to high/low cropping frequencies, crop yields, and returns to labor across sites (Fig. 7). It was also apparent how market influence and population interact to shape the potential variability and predictability of land system outcomes. For example, land-use outcomes in the Laotian sites showed little to no sensitivity to variations in cost and price parameters at observed population density and market influence settings. However, when population density and market influence were experimentally increased, land-use outcomes varied much more widely with alternative cost and price parameter settings. This suggests that population density and market influence may become increasingly important factors in such land systems, and the mechanisms through which agents respond to such forces, for example capital-for-labor substitution and/or multi-cropping, need to be included in future model versions.

### 5.2. Model accuracy versus generality

Some local factors that mediate land per capita ratios and constrain households' livelihood choice sets, such as social networks and land tenure rules, were not represented by the model. The social structures in which individuals are embedded vary widely across land systems, are heavily context dependent, and are not easily generalized [13]. The models presented here are still incomplete. Future efforts will implement processes hypothesized to be important according to the model results reported here. For example, a household-level model will be constructed and compared with the current settlement-level agent representation to systematically test in which contexts a settlement versus household agent representation is necessary, and which additional processes are needed to explain outcomes across different land systems and locations. Although, even the modest amounts of variation in land-use patterns explained by the current model version across different land systems demonstrate the value of this ABM for cross-site comparisons of the causes and consequences of local land-use change globally.

Certainly, over-simplifying the context in which land-use decision-making is embedded can lead to incomplete and/or incorrect understanding of the forces that shape land-use choices. Case studies and models of local land system change provide the in-depth, site-specific knowledge that is invaluable for understanding the local realities of global economic and environmental change. On the other hand, representing the full complexity of social interactions that influence land-use choices runs counter to the aim of understanding more general and larger-scale trends in land system change; the impracticality of acquiring such detailed data across sites, coupled with the limitations of human cognition to navigate such complexity, is prohibitive. Thus, a complementary effort is the pursuit of generalized process and system knowledge to systematically integrate local findings and build theory towards predicting large-scale changes that result from the cumulative effects of local land system changes. As we demonstrate here, a viable way forward in understanding land use as a global change process is by starting with simple models, testing them against current theory and empirical data, and gradually building-in more complexities through an experimental, virtual laboratory approach as needed to better explain and predict observations on the real world.

## Conclusions

A generalized ABM was developed within a virtual laboratory framework for representing cross-scale influences on local land-use decision-making and evaluating model performance across land systems. This ABM was used to explore how agents' decision-making differed in response to the different environmental, demographic, and economic conditions in a set of test sites. When modeled land-use outcomes reasonably approximated those observed in the test sites, the generalized model provided a parsimonious explanation of the major processes structuring observed land-use patterns. Conversely, when the model failed to match real land-use patterns, it did so systematically, providing a common model structure to compare sources of failure and the influence of different structuring processes across sites. Particularly, the applicability of the underlying labor- and risk-minimizing decision-making framework was limited in land systems driven primarily by market forces, which indicated the conditions under which alternative decision-making frameworks are necessary.

Cross-site comparison and synthesis has been identified as a priority by the LCS community [10]. Agent-based model comparison, in particular, has the potential to provide insights into commonalities and differences in decision-making across land systems [20]. The modeling framework presented here formalizes the mechanisms underlying land-use and livelihood decisions, which makes it possible to examine agents' adaptive responses to local contextual and large-scale forces. Furthermore, doing so with a generic model structure provides a means for systematic comparisons of decision-making processes across land systems and contributes to our understanding of patterns of local land-use and livelihood changes globally. Future implementations of this agent-based virtual laboratory approach will test hypotheses of how and under what conditions driving forces of land system change might differ from the generic model across a wider, more representative range of land systems, and how agents' motivations might change as economic globalization restructures local economic opportunities.

## Supporting Information

Figure S1 **Site characteristics and agent labor allocation.** (a) Comparison of counts per land-use/cover category between real (blue) and modeled (red) landscapes, (b) model representation of sample site landscape and (c) land suitability, and (d) the average percentage across agents of labor allocated to (from top to bottom) subsistence farm, market-oriented farm, and non-farm wage (NFW) labor.
(TIF)

Figure S2 **Measures of model error.** (a) Relationship between distance and Spearman's Rho for landscape-level, aggregate land-use/cover category counts in each experimental combination; (b) distance measure of the landscape-level, aggregate differences in land-use/cover category counts between the real and modeled (colored points) and null (black line) landscapes; (c) distance measure of aggregate difference in counts of landscape cells in land-use/cover categories per counts of landscape cells in each land suitability class between real and modeled (colored points) and null (black line) landscapes.
(TIF)

Figure S3 **Site characteristics and agent labor allocation.** (a) Comparison of counts per land-use/cover category between real (blue) and modeled (red) landscapes, (b) model representation of sample site landscape and (c) land suitability, and (d) the average percentage across agents of labor allocated to (from top to bottom) subsistence farm, market-oriented farm, and non-farm wage (NFW) labor.
(TIF)

Figure S4 **Measures of model error.** (a) Relationship between distance and Spearman's Rho for landscape-level, aggregate land-use/cover category counts in each experimental combination; (b) distance measure of the landscape-level, aggregate differences in land-use/cover category counts between the real and modeled (colored points) and null (black line) landscapes; (c) distance measure of aggregate difference in counts of landscape cells in land-use/cover categories per counts of landscape cells in each land suitability class between real and modeled (colored points) and null (black line) landscapes.
(TIF)

Figure S5 **Site characteristics and agent labor allocation.** (a) Comparison of counts per land-use/cover category between real (blue) and modeled (red) landscapes, (b) model representation of sample site landscape and (c) land suitability, and (d) the average percentage across agents of labor allocated to (from top to bottom) subsistence farm, market-oriented farm, and non-farm wage (NFW) labor.
(TIF)

Figure S6 **Measures of model error.** (a) Relationship between distance and Spearman's Rho for landscape-level, aggregate land-use/cover category counts in each experimental combination; (b) distance measure of the landscape-level, aggregate differences in land-use/cover category counts between the real and modeled (colored points) and null (black line) landscapes; (c) distance measure of aggregate difference in counts of landscape cells in land-use/cover categories per counts of landscape cells in each land suitability class between real and modeled (colored points) and null (black line) landscapes.
(TIF)

Figure S7 **Site characteristics and agent labor allocation.** (a) Comparison of counts per land-use/cover category between real (blue) and modeled (red) landscapes, (b) model representation of sample site landscape and (c) land suitability, and (d) the average percentage across agents of labor allocated to (from top to bottom) subsistence farm, market-oriented farm, and non-farm wage (NFW) labor.
(TIF)

Figure S8 **Measures of model error.** (a) Relationship between distance and Spearman's Rho for landscape-level, aggregate land-use/cover category counts in each experimental combination; (b) distance measure of the landscape-level, aggregate differences in land-use/cover category counts between the real and modeled (colored points) and null (black line) landscapes; (c) distance measure of aggregate difference in counts of landscape cells in land-use/cover categories per counts of landscape cells in each land suitability class between real and modeled (colored points) and null (black line) landscapes.
(TIF)

Figure S9 **Site characteristics and agent labor allocation.** (a) Comparison of counts per land-use/cover category between real (blue) and modeled (red) landscapes, (b) model representation of sample site landscape and (c) land suitability, and (d) the average percentage across agents of labor allocated to (from top to bottom) subsistence farm, market-oriented farm, and non-farm wage (NFW) labor.
(TIF)

Figure S10 **Measures of model error.** (a) Relationship between distance and Spearman's Rho for landscape-level, aggregate land-use/cover category counts in each experimental combination; (b) distance measure of the landscape-level, aggregate differences in land-use/cover category counts between the real and modeled (colored points) and null (black line) landscapes; (c) distance measure of aggregate difference in counts of landscape cells in land-use/cover categories per counts of landscape cells in each land suitability class between real and modeled (colored points) and null (black line) landscapes.
(TIF)

Figure S11 **Site characteristics and agent labor allocation.** (a) Comparison of counts per land-use/cover category between real (blue) and modeled (red) landscapes, (b) model representation of sample site landscape and (c) land suitability, and (d) the average percentage across agents of labor allocated to (from top to bottom) subsistence farm, market-oriented farm, and non-farm wage (NFW) labor.
(TIF)

Figure S12 **Measures of model error.** (a) Relationship between distance and Spearman's Rho for landscape-level, aggregate land-use/cover category counts in each experimental combination; (b) distance measure of the landscape-level, aggregate differences in land-use/cover category counts between the real and modeled (colored points) and null (black line) landscapes; (c) distance measure of aggregate difference in counts of landscape cells in land-use/cover categories per counts of landscape cells in each land suitability class between real and modeled (colored points) and null (black line) landscapes.
(TIF)

Supplementary Information S1 Section S1, Site-specific statistical analyses. Error measurements calculated in Eq. 1 are disaggregated by land suitability class to evaluate differences between observed and modeled land-use/cover outcomes. Section S2, Site-specific descriptions and results. Geographic descriptions of study sites and site-specific statistics evaluating the agreement between modeled, null model, and real land-use/cover outcomes.
(DOCX)
