# Supplementary figures and images for: Cross-Site Comparison of Land-Use Decision-Making and Its Consequences across Land Systems with a Generalized Agent-Based Model
Source: PLoS One. 2014 Jan 29;9(1):e86179. doi: 10.1371/journal.pone.0086179 (PMC3906050; doi:10.1371/journal.pone.0086179)

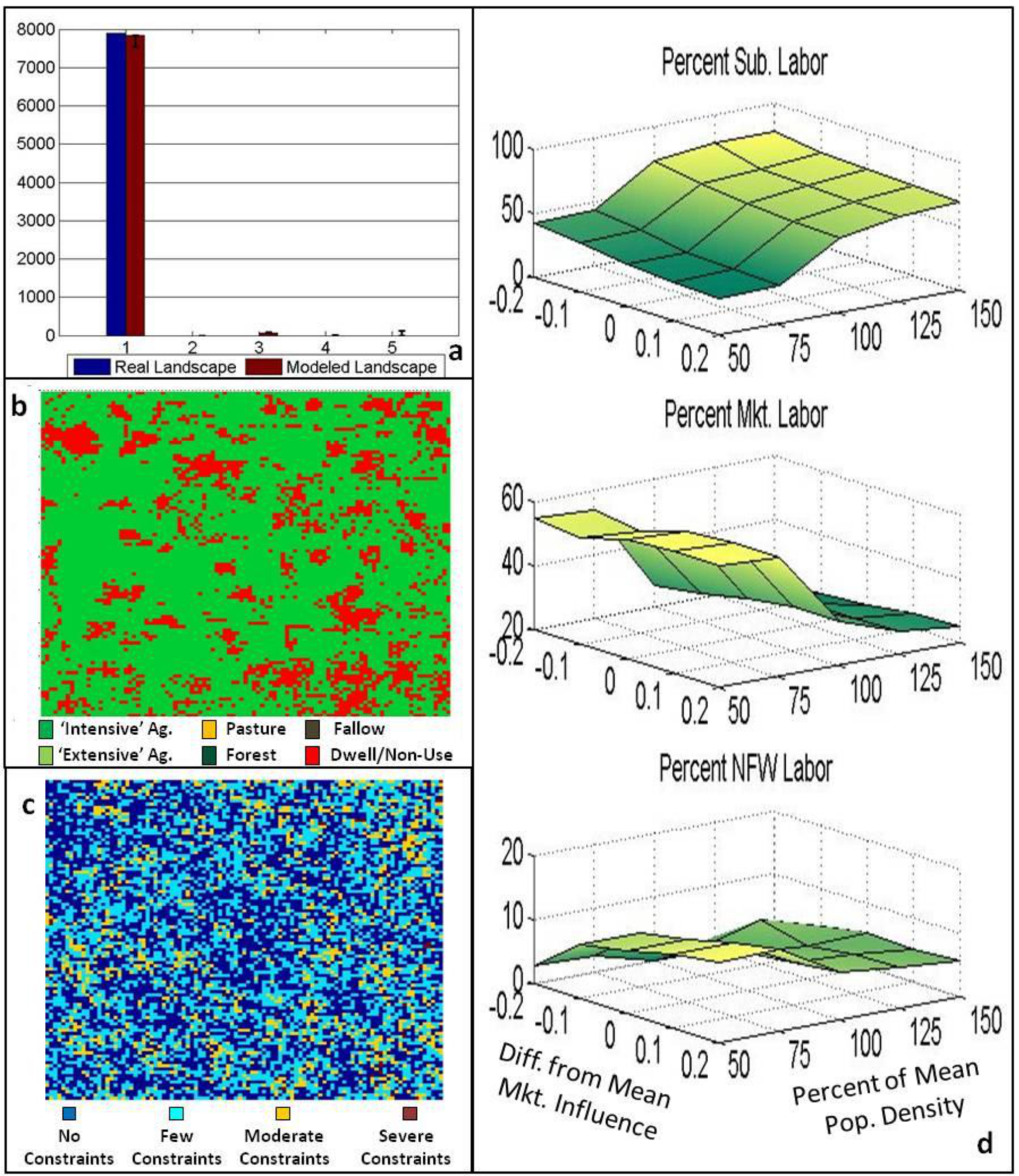

Supplement: Figure S1 — Site characteristics and agent labor allocation. (a) Comparison of counts per land-use/cover category between real (blue) and modeled (red) landscapes, (b) model representation of sample site landscape and (c) land suitability, and (d) the average percentage across agents of labor allocated to (from top to bottom) subsistence farm, market-oriented farm, and non-farm wage (NFW) labor. (TIF) [file pone.0086179.s001.tif]

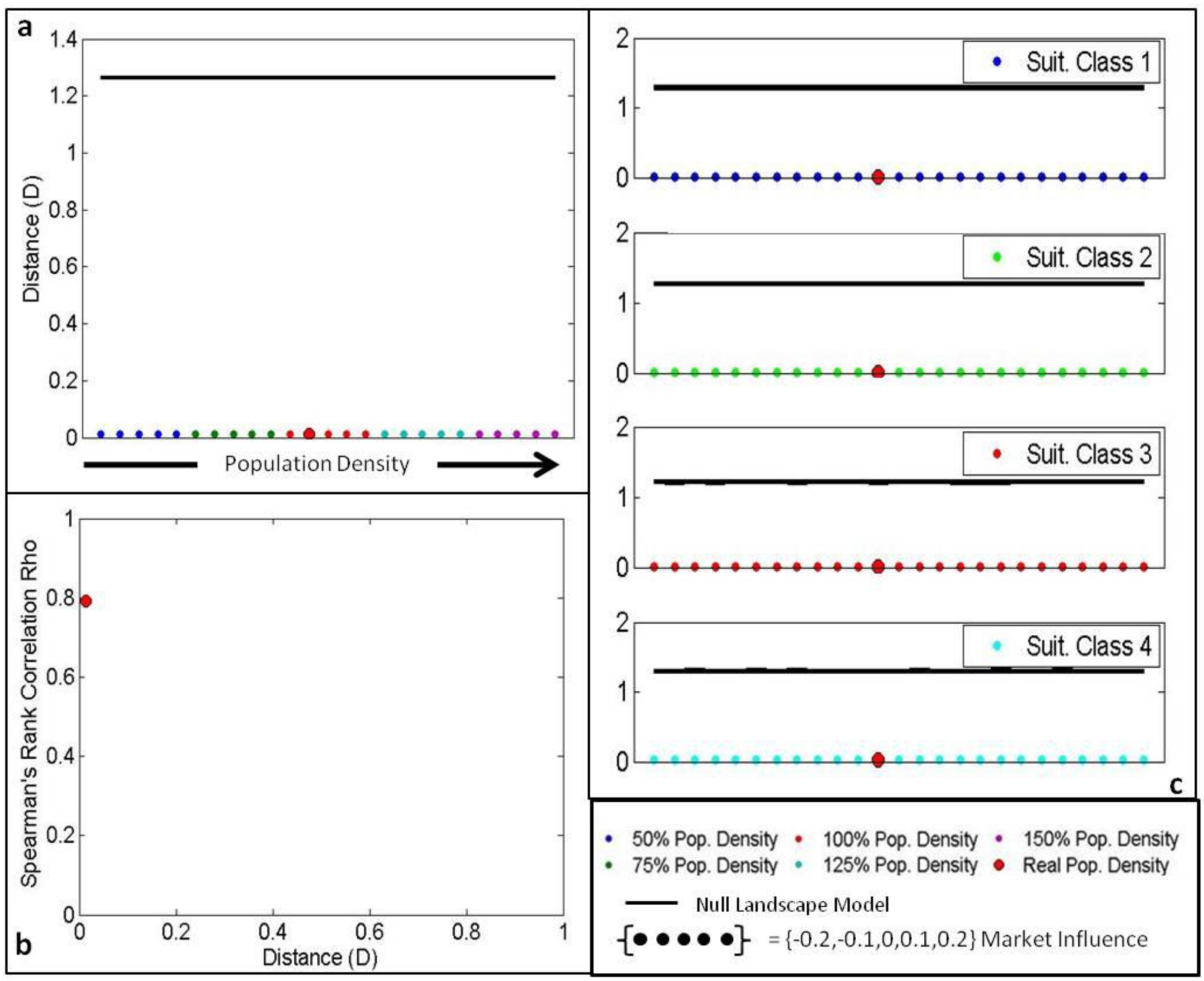

Supplement: Figure S2 — Measures of model error. (a) Relationship between distance and Spearman's Rho for landscape-level, aggregate land-use/cover category counts in each experimental combination; (b) distance measure of the landscape-level, aggregate differences in land-use/cover category counts between the real and modeled (colored points) and null (black line) landscapes; (c) distance measure of aggregate difference in counts of landscape cells in land-use/cover categories per counts of landscape cells in each land suitability class between real and modeled (colored points) and null (black line) landscapes. (TIF) [file pone.0086179.s002.tif]

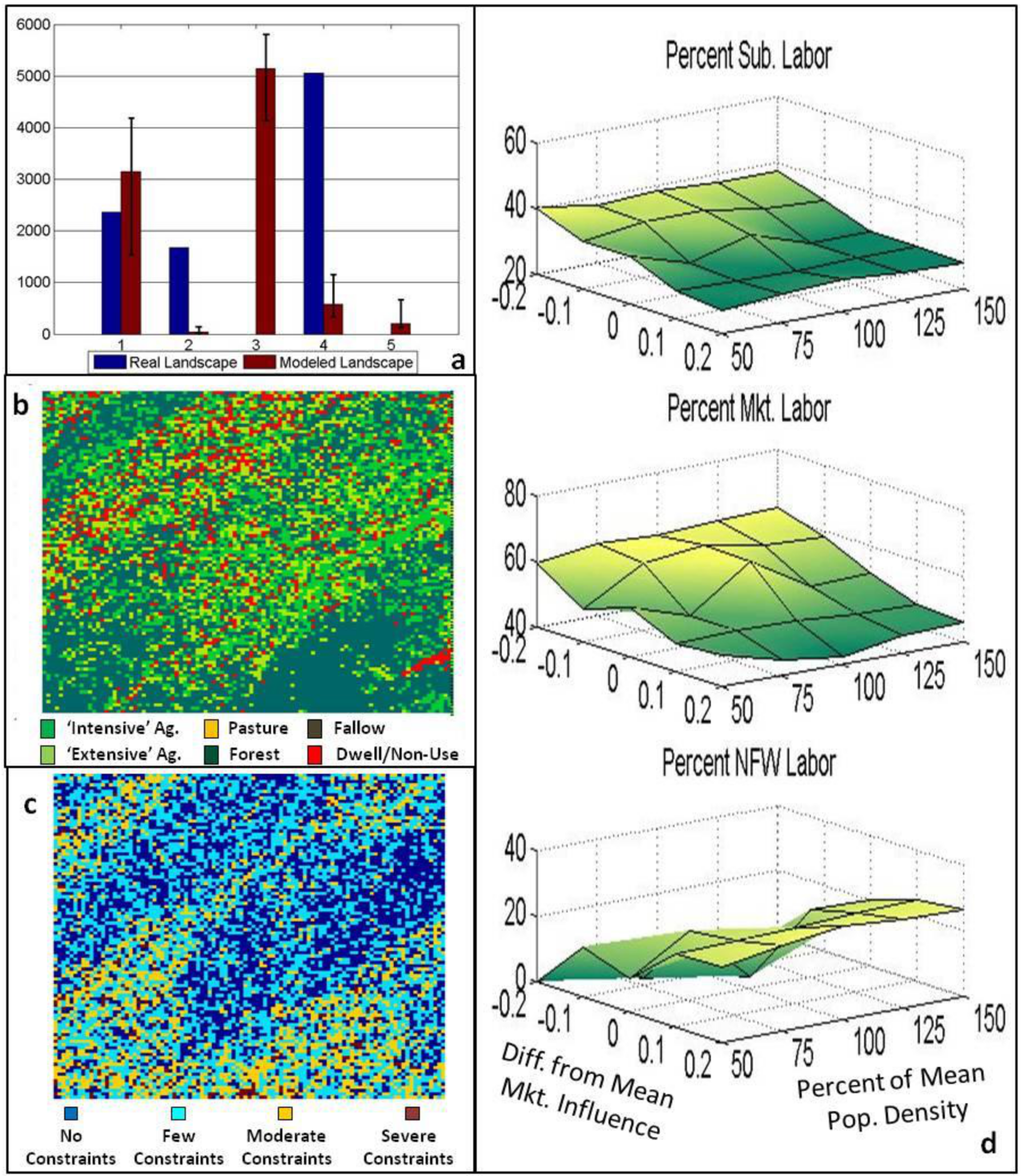

Supplement: Figure S3 — Site characteristics and agent labor allocation. (a) Comparison of counts per land-use/cover category between real (blue) and modeled (red) landscapes, (b) model representation of sample site landscape and (c) land suitability, and (d) the average percentage across agents of labor allocated to (from top to bottom) subsistence farm, market-oriented farm, and non-farm wage (NFW) labor. (TIF) [file pone.0086179.s003.tif]

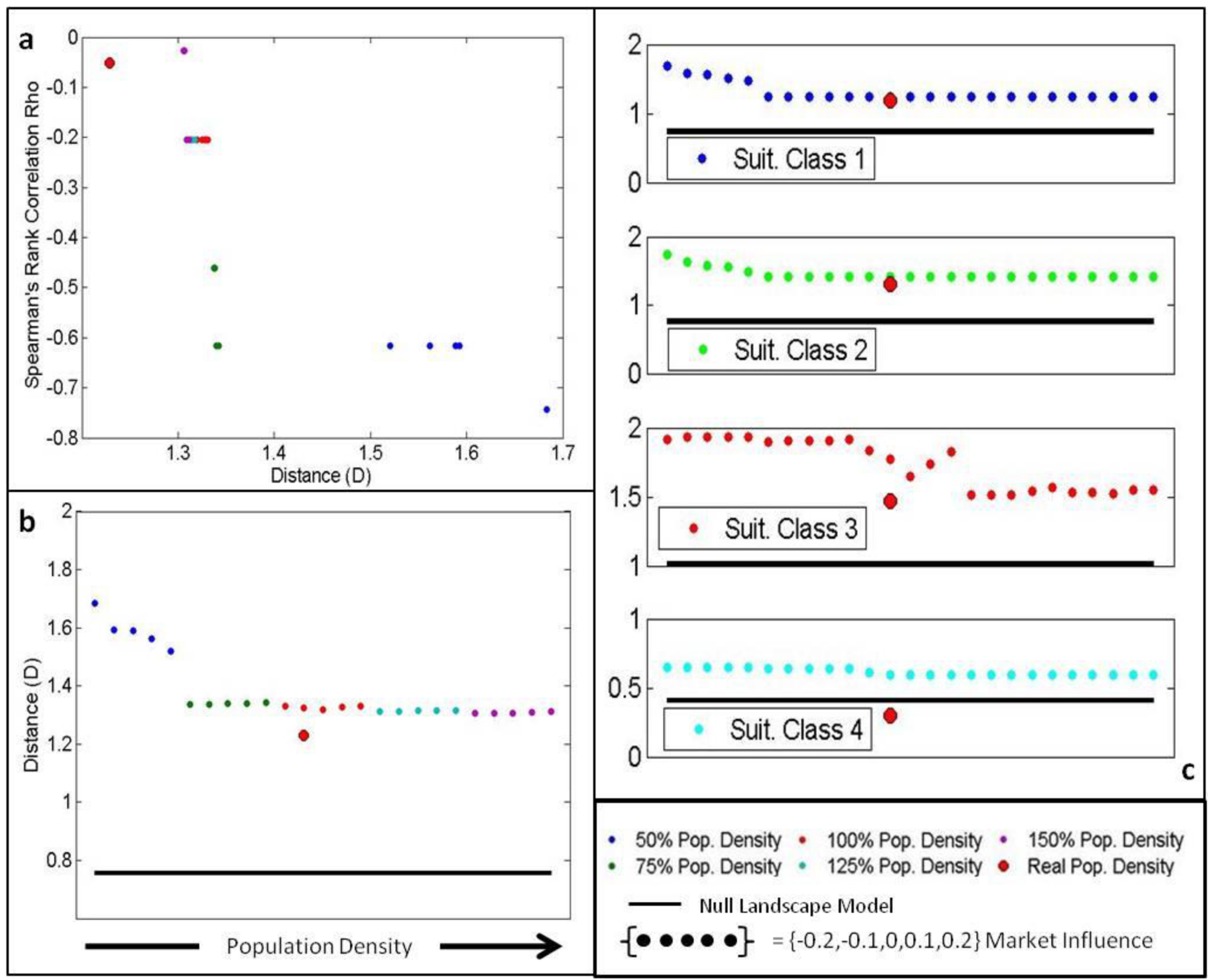

Supplement: Figure S4 — Measures of model error. (a) Relationship between distance and Spearman's Rho for landscape-level, aggregate land-use/cover category counts in each experimental combination; (b) distance measure of the landscape-level, aggregate differences in land-use/cover category counts between the real and modeled (colored points) and null (black line) landscapes; (c) distance measure of aggregate difference in counts of landscape cells in land-use/cover categories per counts of landscape cells in each land suitability class between real and modeled (colored points) and null (black line) landscapes. (TIF) [file pone.0086179.s004.tif]

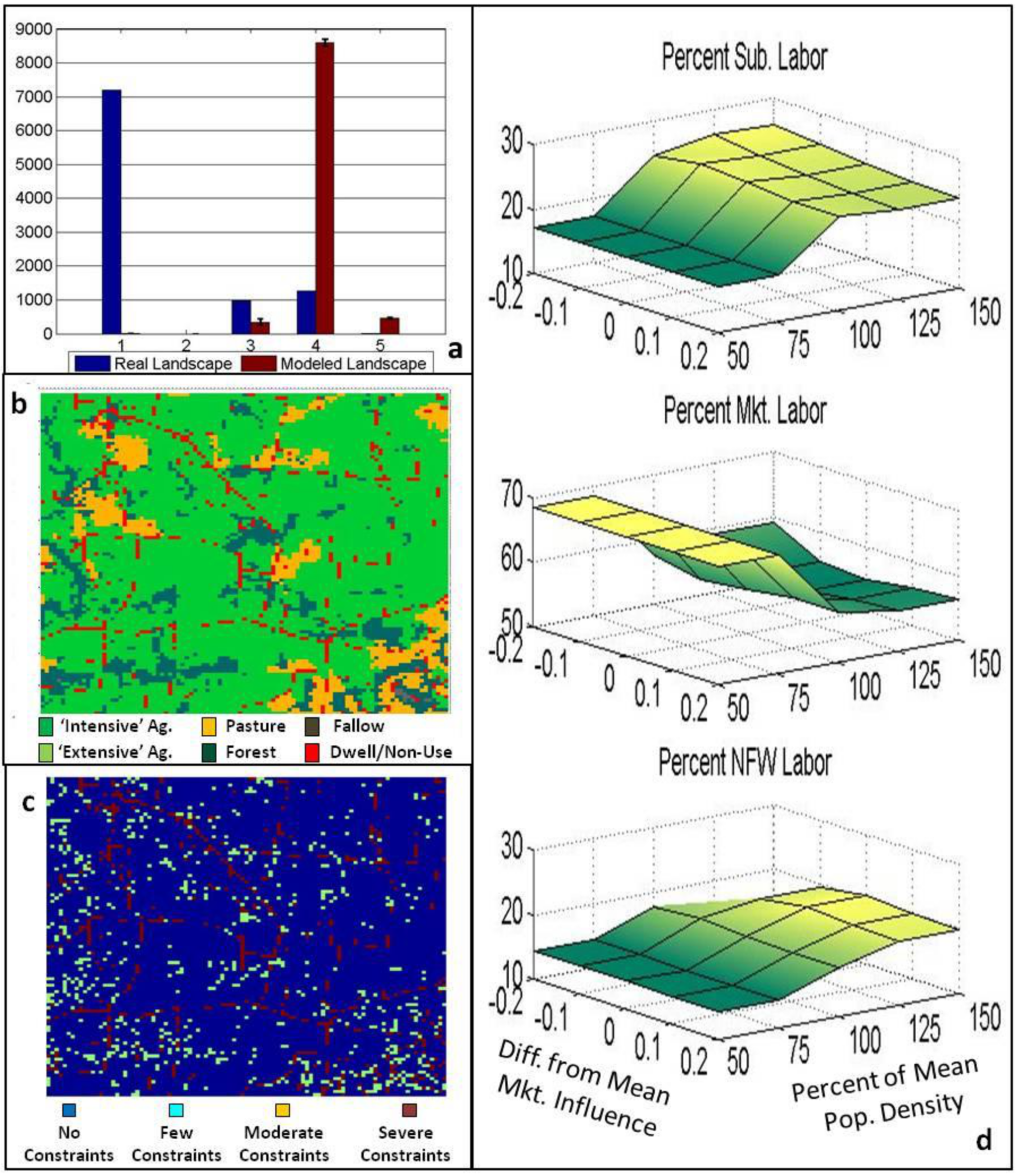

Supplement: Figure S5 — Site characteristics and agent labor allocation. (a) Comparison of counts per land-use/cover category between real (blue) and modeled (red) landscapes, (b) model representation of sample site landscape and (c) land suitability, and (d) the average percentage across agents of labor allocated to (from top to bottom) subsistence farm, market-oriented farm, and non-farm wage (NFW) labor. (TIF) [file pone.0086179.s005.tif]

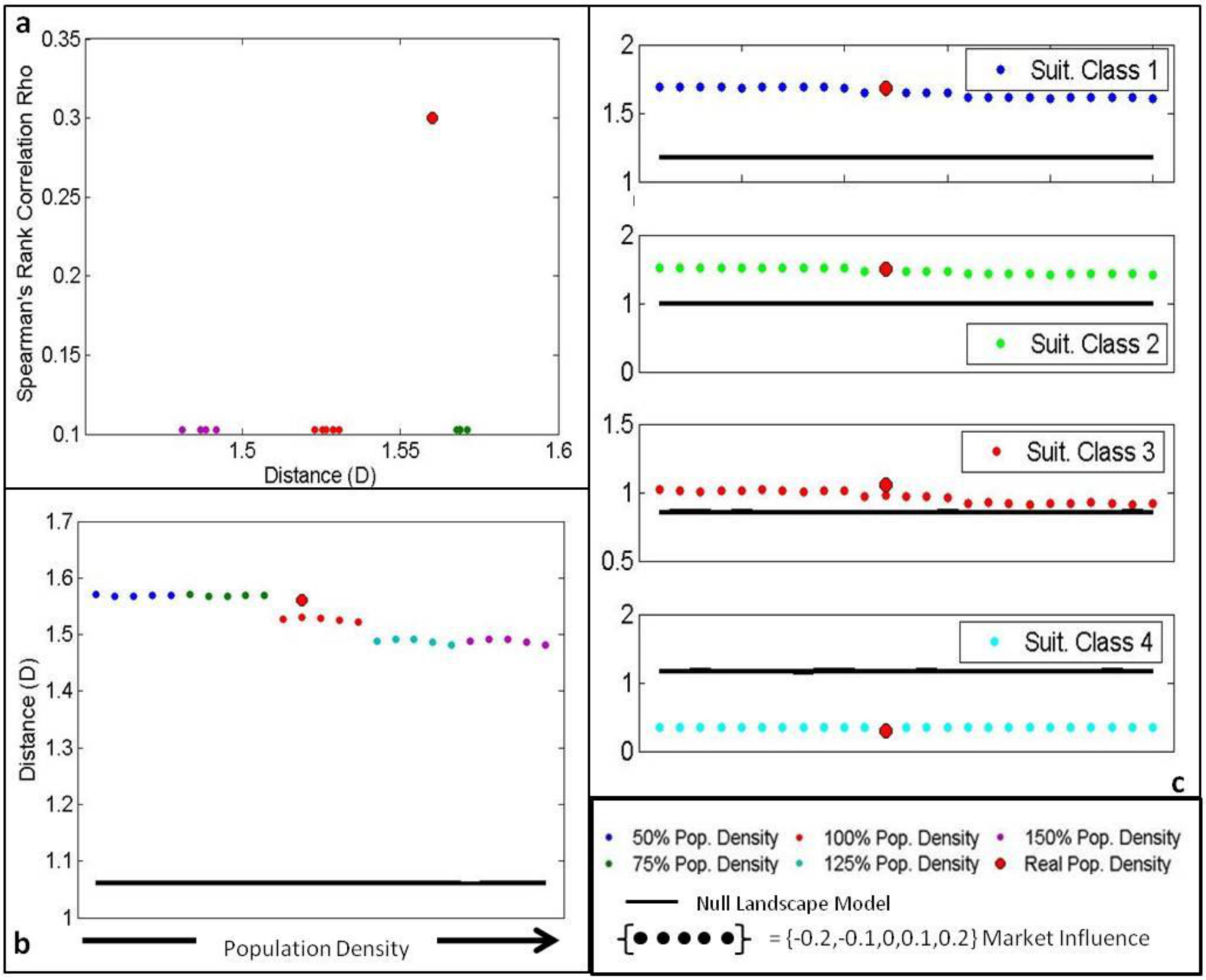

Supplement: Figure S6 — Measures of model error. (a) Relationship between distance and Spearman's Rho for landscape-level, aggregate land-use/cover category counts in each experimental combination; (b) distance measure of the landscape-level, aggregate differences in land-use/cover category counts between the real and modeled (colored points) and null (black line) landscapes; (c) distance measure of aggregate difference in counts of landscape cells in land-use/cover categories per counts of landscape cells in each land suitability class between real and modeled (colored points) and null (black line) landscapes. (TIF) [file pone.0086179.s006.tif]

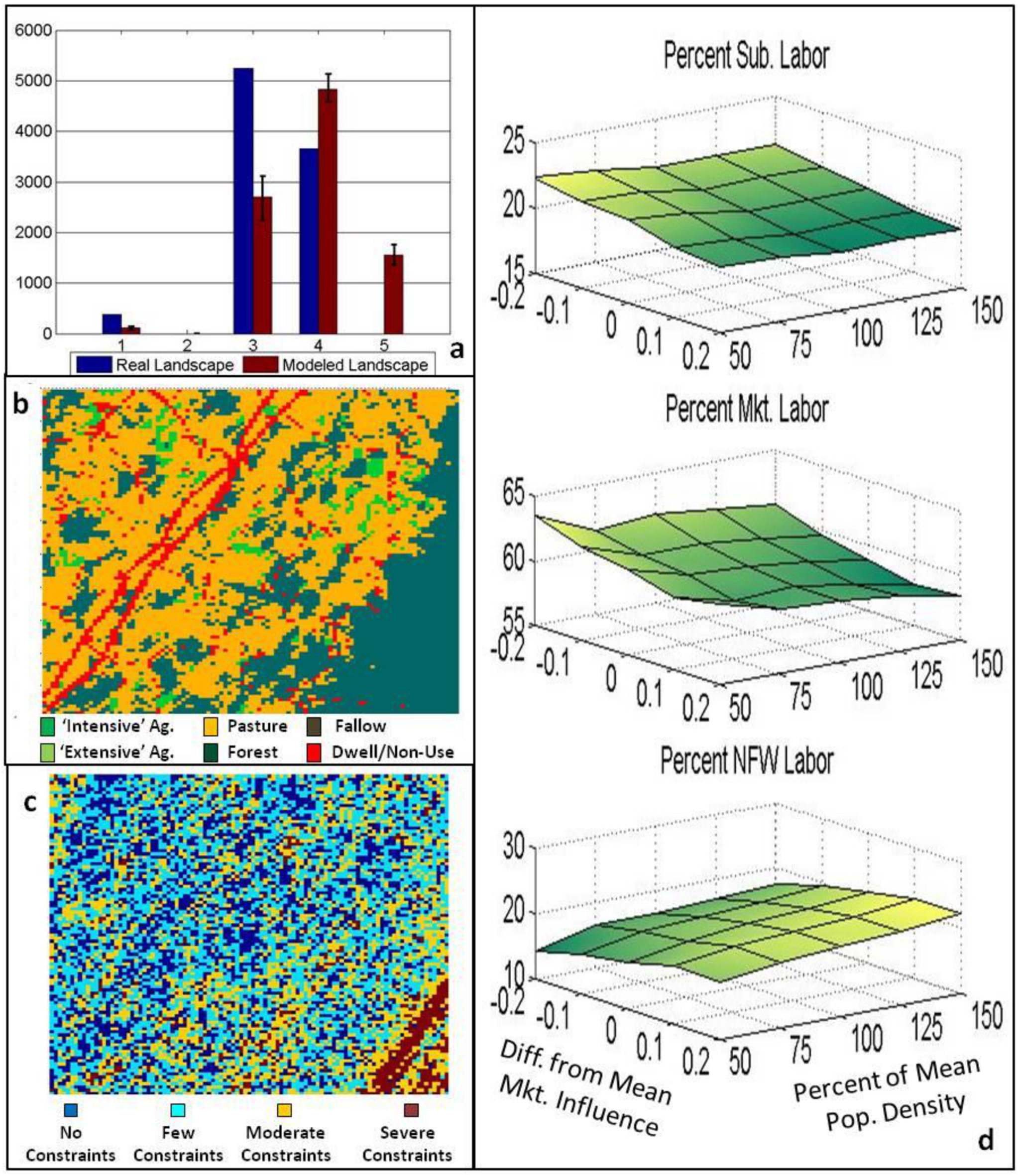

Supplement: Figure S7 — Site characteristics and agent labor allocation. (a) Comparison of counts per land-use/cover category between real (blue) and modeled (red) landscapes, (b) model representation of sample site landscape and (c) land suitability, and (d) the average percentage across agents of labor allocated to (from top to bottom) subsistence farm, market-oriented farm, and non-farm wage (NFW) labor. (TIF) [file pone.0086179.s007.tif]

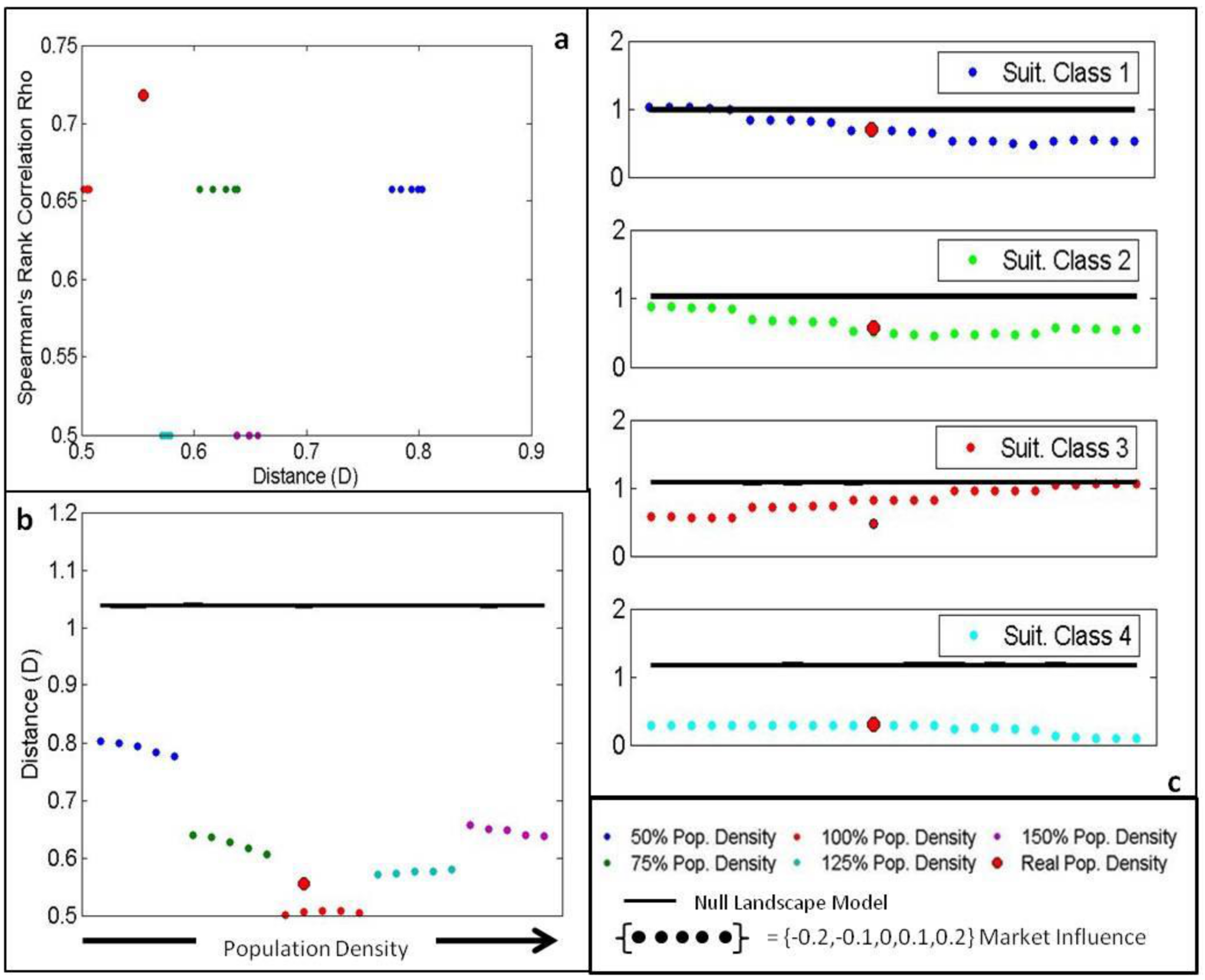

Supplement: Figure S8 — Measures of model error. (a) Relationship between distance and Spearman's Rho for landscape-level, aggregate land-use/cover category counts in each experimental combination; (b) distance measure of the landscape-level, aggregate differences in land-use/cover category counts between the real and modeled (colored points) and null (black line) landscapes; (c) distance measure of aggregate difference in counts of landscape cells in land-use/cover categories per counts of landscape cells in each land suitability class between real and modeled (colored points) and null (black line) landscapes. (TIF) [file pone.0086179.s008.tif]

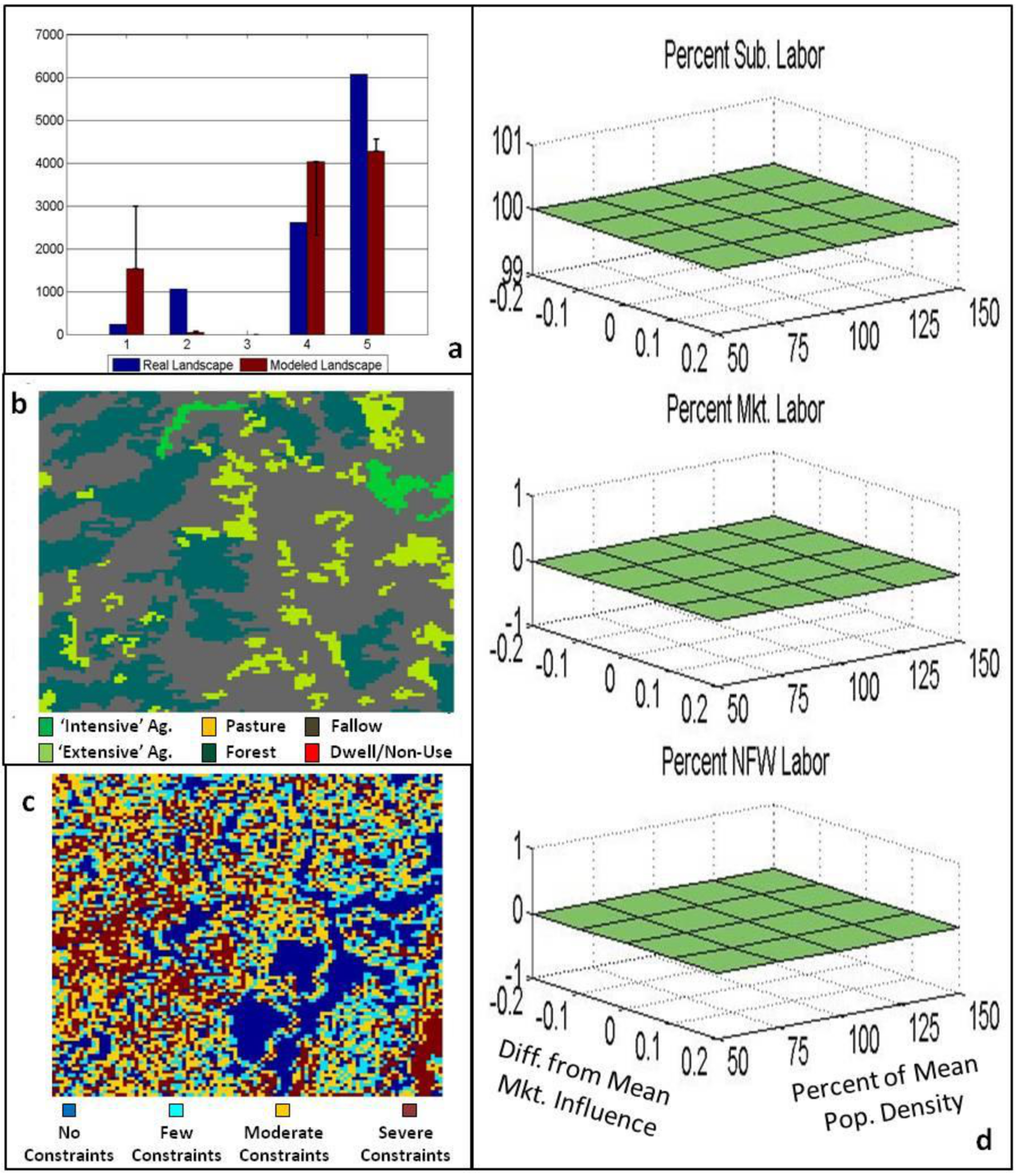

Supplement: Figure S9 — Site characteristics and agent labor allocation. (a) Comparison of counts per land-use/cover category between real (blue) and modeled (red) landscapes, (b) model representation of sample site landscape and (c) land suitability, and (d) the average percentage across agents of labor allocated to (from top to bottom) subsistence farm, market-oriented farm, and non-farm wage (NFW) labor. (TIF) [file pone.0086179.s009.tif]

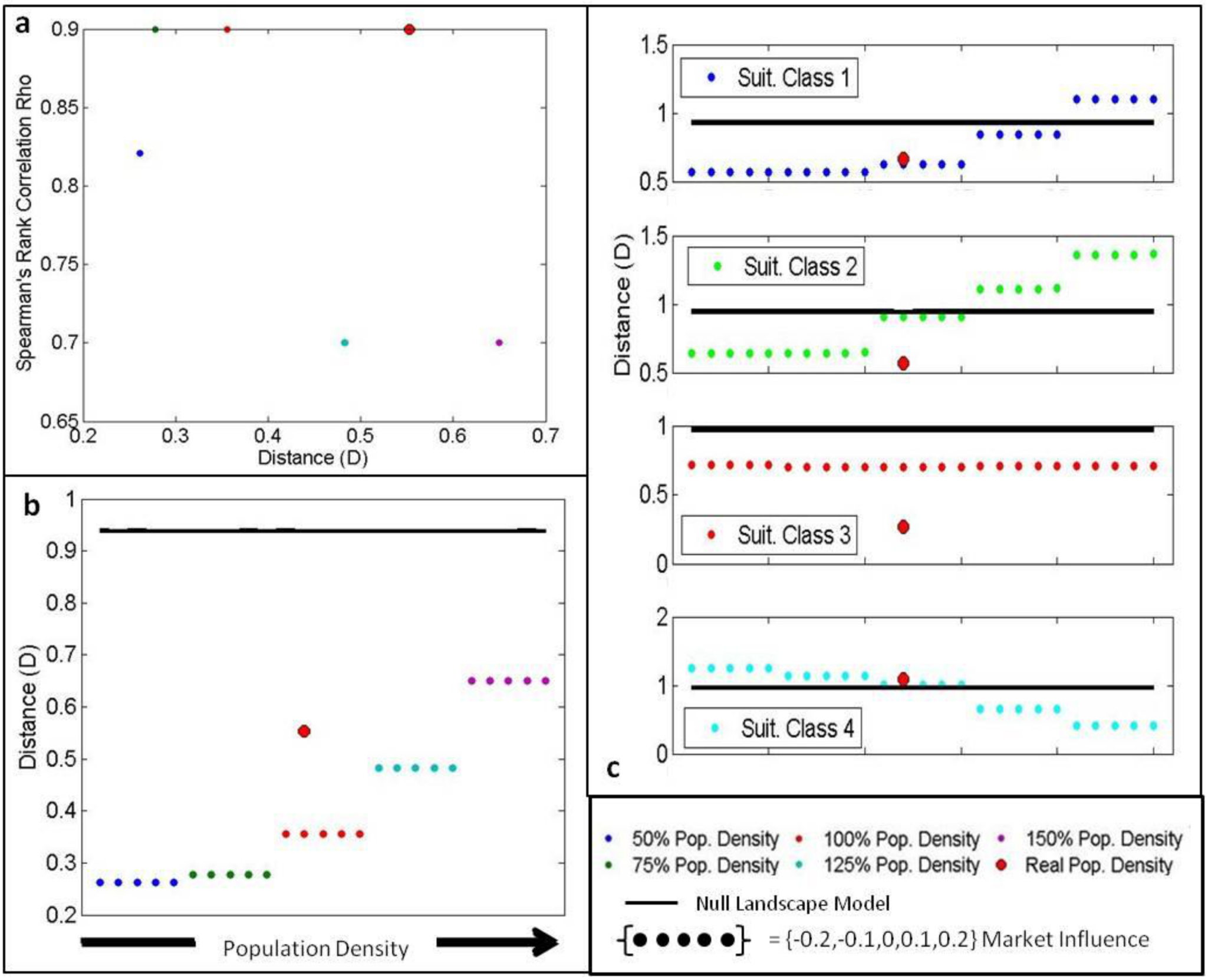

Supplement: Figure S10 — Measures of model error. (a) Relationship between distance and Spearman's Rho for landscape-level, aggregate land-use/cover category counts in each experimental combination; (b) distance measure of the landscape-level, aggregate differences in land-use/cover category counts between the real and modeled (colored points) and null (black line) landscapes; (c) distance measure of aggregate difference in counts of landscape cells in land-use/cover categories per counts of landscape cells in each land suitability class between real and modeled (colored points) and null (black line) landscapes. (TIF) [file pone.0086179.s010.tif]

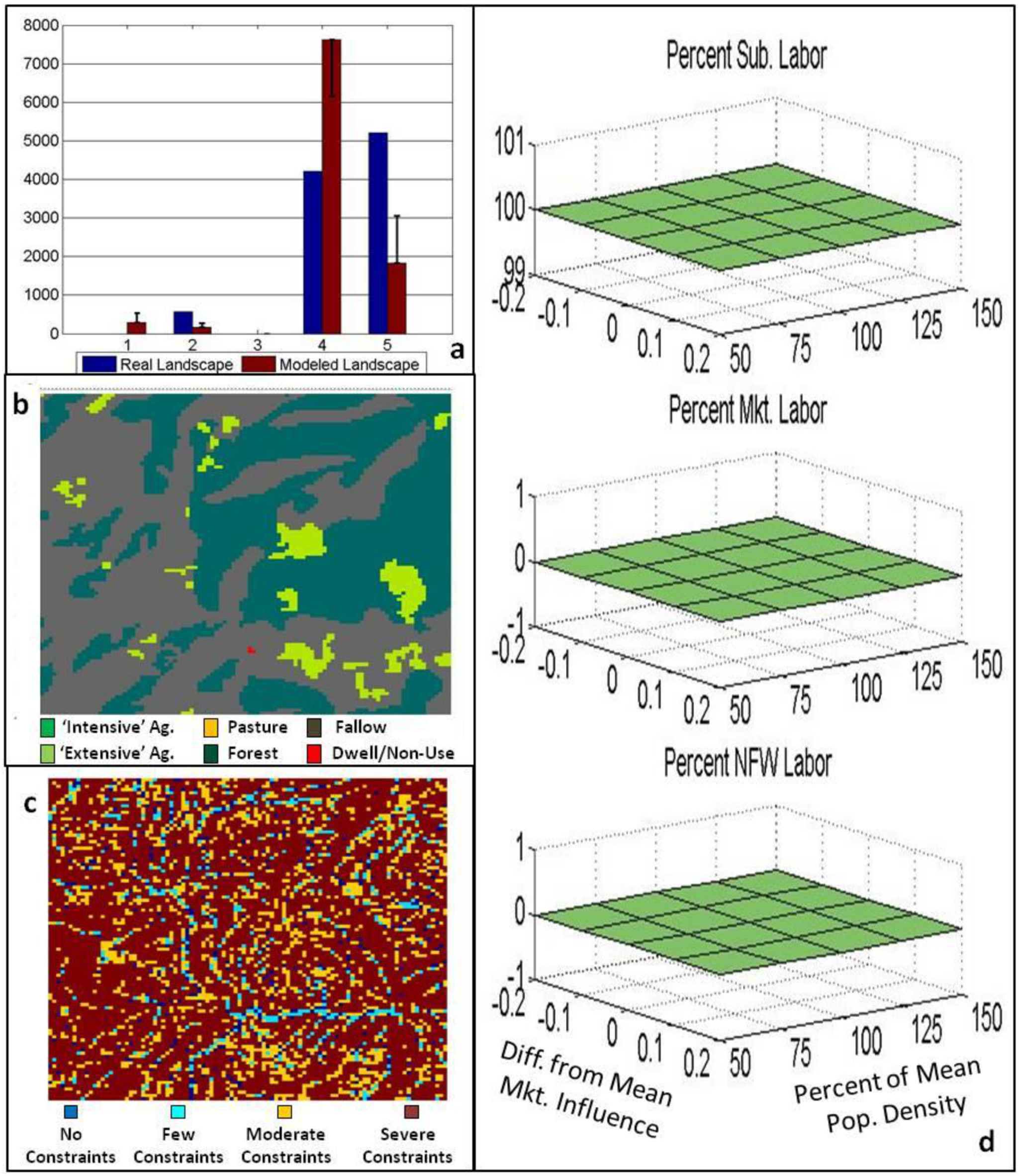

Supplement: Figure S11 — Site characteristics and agent labor allocation. (a) Comparison of counts per land-use/cover category between real (blue) and modeled (red) landscapes, (b) model representation of sample site landscape and (c) land suitability, and (d) the average percentage across agents of labor allocated to (from top to bottom) subsistence farm, market-oriented farm, and non-farm wage (NFW) labor. (TIF) [file pone.0086179.s011.tif]

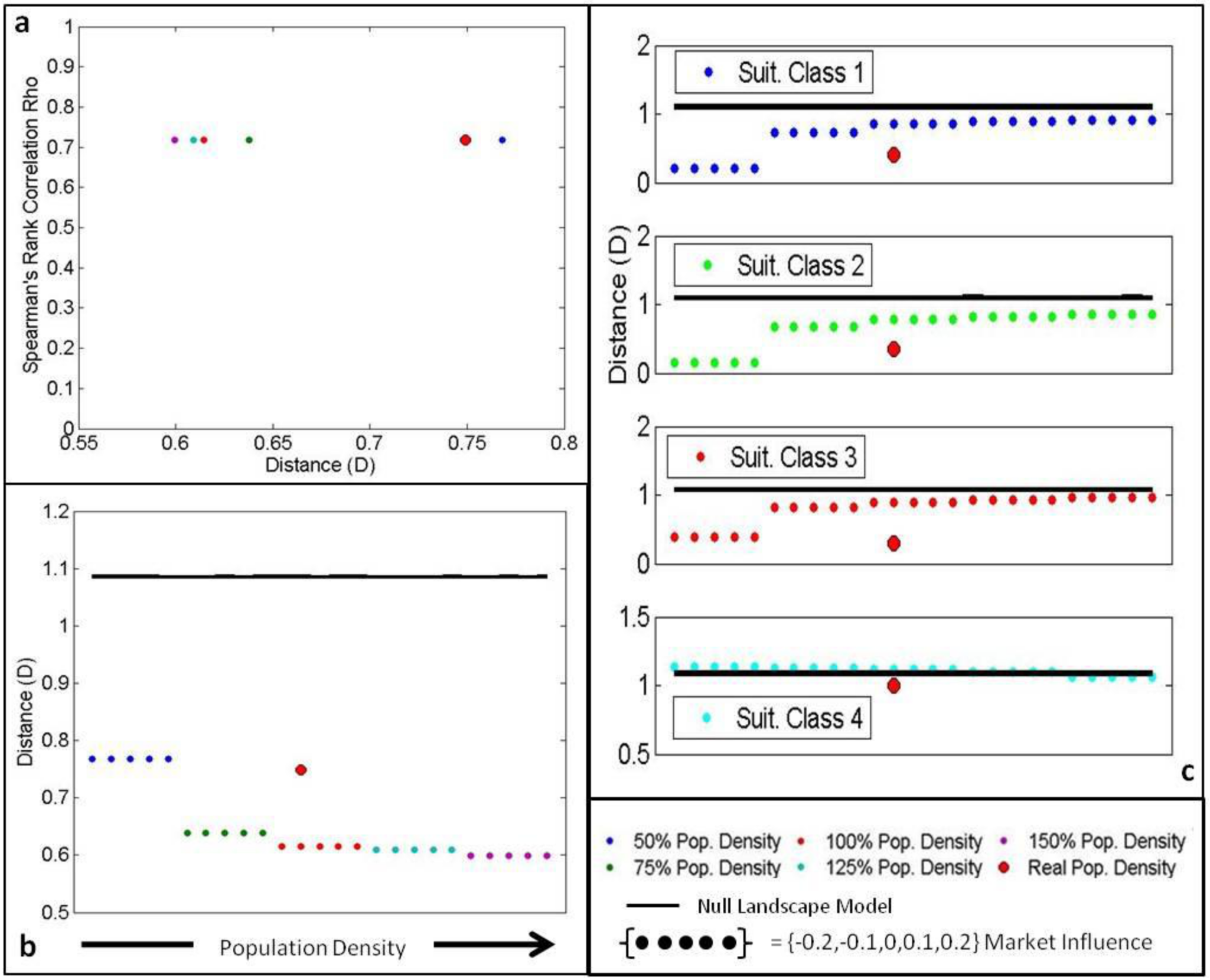

Supplement: Figure S12 — Measures of model error. (a) Relationship between distance and Spearman's Rho for landscape-level, aggregate land-use/cover category counts in each experimental combination; (b) distance measure of the landscape-level, aggregate differences in land-use/cover category counts between the real and modeled (colored points) and null (black line) landscapes; (c) distance measure of aggregate difference in counts of landscape cells in land-use/cover categories per counts of landscape cells in each land suitability class between real and modeled (colored points) and null (black line) landscapes. (TIF) [file pone.0086179.s012.tif]
