# Supplementary material for: Cross-Site Comparison of Land-Use Decision-Making and Its Consequences across Land Systems with a Generalized Agent-Based Model
Source: PLoS One. 2014 Jan 29;9(1):e86179. doi: 10.1371/journal.pone.0086179 (PMC3906050; doi:10.1371/journal.pone.0086179)
Supplement: Supplementary Information S1 — Section S1, Site-specific statistical analyses. Error measurements calculated in Eq. 1 are disaggregated by land suitability class to evaluate differences between observed and modeled land-use/cover outcomes. Section S2, Site-specific descriptions and results. Geographic descriptions of study sites and site-specific statistics evaluating the agreement between modeled, null model, and real land-use/cover outcomes. (DOCX) [file pone.0086179.s013.docx]

**Supplementary Information**

**S1. Site-Specific Analyses**

S1.1. Site-Specific Statistical Analyses

Disaggregating Eq. 1 into land-use/cover within each land suitability class provides a more nuanced assessment of model error, and a measure that could be compared across land-use systems of widely varying composition. Within each of the four land suitability classes (1 = most suitable for agriculture to 4 = least suitable for agriculture), the ratio of the number of cells in each land use/cover category to the number of cells in the land suitability class is calculated (i.e. percent of area in each land suitability class in each land-use/cover category). These ratios are compared to those from the real landscape by modifying Eq. 1 to obtain a distance measure, *DS*, for each land suitability class:

$DS_{ic}=\sum_{i=1}^{5} \sqrt{\left(RS_{ic}-MS_{ic}\right)^{2}}$ ; (S-1)

where *RS_ic_* and *MS_ic_* are ratios of land-use/cover cells to land suitability class cells in the real and modeled landscapes (including null), respectively, for land use/cover category *i* in land suitability class, *c*.

The distributions of each land-use/cover category within the real and modeled landscapes are compared using Shannon's Evenness Index (SEI).

$SEI=-\frac{\sum_{i=1}^{n} p_{i}\log(p_{i})}{\log(n)}$ ; (S-2)

where *p* is the proportion of the landscape in land-use/cover category, *i*, and *n* is the number of potential categories tested (equal to 5). SEI provides a description of how evenly distributed or biased land-uses/covers are within the landscape, and whether land-use/cover composition is predicted well by a null model (i.e. each land-use/cover class was roughly equally represented). SEI provides useful insight into how a modeled landscape might differ from a real landscape when the other measures indicated poor model performance.

**S2. Site-Specific Descriptions and Results**

S2.1. Western Shandong Province, China

This site was located in the prime agricultural areas of the North China Plain near the town of Liaocheng in Shandong Province. Global environmental, demographic, and market data was re-sampled using the WGS 1984 UTM Zone 50N projection. Classified LandSat images from Ellis et al. [1] were used as input land cover layers.

*S2.1.1. Results*

Variations in land-use/cover outcomes due to experimental model settings were minimal. All market influence-population density combinations produced the same rankings of land-use/cover categories and negligible variations in the difference between modeled and real counts (Fig. S1). The model produced aggregate land use/cover category counts at the landscape-level that closely matched those observed in the real landscape, and all experimental settings performed better than the null model (Fig. S1). Similarly, the distribution of counts among land-use/cover categories, measured using SEI, was very close to that of the real landscape (Fig. S2). Consequently, disaggregating land-use/cover counts to land suitability classes and specific land-uses/covers demonstrated superior model performance compared to the null model across all land suitability classes (Fig. S2). Minor model errors occurred from the introduction of small amounts of pasture use, which was not seen in the real landscape. These errors produced the largest variation in landscape outcomes, and occurred when market influence and population density settings were both below mean values of the real landscape (Fig. S2).

S2.2. Northern Hunan Province, China

This site was located in the hilly regions of Hunan Province in south central China. Global environmental, demographic, and market data was re-sampled using the WGS 1984 UTM Zone 49N projection. Classified LandSat images from Ellis et al. [1] were used as input land cover layers.

*S2.2.1. Results*

The aggregate distance in land-use/cover category counts between the real and modeled landscapes was relatively large (Fig. S3). The spatially explicit representation of population density and market influence performed best, and model errors generally increased as population density decreased. Similarly, zero to negative rho values in category ranking were seen (Fig. S3). Disaggregating these results based on land suitability class demonstrated similar model failures (Fig. S3). The null model performed consistently better across land suitability classes, except for the spatially explicit model setting produced a better agreement with land-use/cover counts observed in land suitability class 4 in the real landscape. In contrast, the distribution of land-use/cover category counts measured by SEI was more similar to the real landscape than the null model for all experimental setting (Fig. S4). Real and modeled land-use/cover distributions became increasingly similar as experimental settings approached values equal to the real landscape, with the distribution generated by the spatially explicit representation nearly identical to the real landscape. Finally, variability in land-use outcomes was high within and across experimental settings, as indicated by relatively large inter-quartile ranges in distributions of errors in land-use/cover category counts (Fig. S4).

*S2.3 Western Kentucky, U.S.A.*

This site was located in prime agricultural areas along the border of Kentucky and Tennessee. Global environmental, demographic, and market data was re-sampled using the WGS 1984 UTM Zone 16N projection. Classified LandSat images from 2001 NLCD [3] were used as input land cover layers.

*S2.3.1. Results*

The model failed to produce counts of most land-use/cover categories accurately (Fig. S5). The null model produced land-use outcomes more similar to the real landscape. Of all the experimental model settings, the ranking of each land-use/cover category was best for the spatially explicit representation of population density and market influence, but the correlation was very weak. Land-use outcomes per land suitability class generally improved as population densities increased due to expansion of pasture (Fig. S5). However, the null model provided a better prediction of land-use/cover counts across land suitability classes 1-3. Although the model out-performed the null outcome for land suitability class 4, this only constituted 0.6 percent of the landscape. SEI also improved as population density increased, and overall the modeled out-performed the null outcome indicating that model errors were systematic rather than random (Fig. S6). Land-use outcomes were most consistent with high market influence and low population density settings (Fig. S6). As population density increased and/or market influence decreased, land-use outcomes became more variable. Finally, the genetic algorithm did not find any successful solutions that could produce all three smallholder behavioral patterns simultaneously.

S2.4. Northwestern Virginia, U.S.A.

This site was located in the Massanutten Mountains near Harrisionburg, VA. Global environmental, demographic, and market data was re-sampled using the WGS 1984 UTM Zone 17N projection. Classified LandSat images from 2001 NLCD [3] were used as input land cover layers.

*S2.4.1 Results*

Modeled land-use outcomes showed a clear pattern of improving as experimental settings approached mean population density and market influence values of the real landscape . A positive correlation was produced between the ranking of real and modeled land-use/cover categories (Fig. S7), and differences between real and modeled category counts were smaller than those between the real and null landscapes (Fig. S7). Disaggregating land-use/cover counts by land suitability classes demonstrated that model errors were generally reduced as population density increased, and market influence had a negligible influence on results within any given population density setting (Fig. S7). The model produced distributions of land-use/cover categories more similar to the real landscape than the null model (Fig. S8).

Trends in model errors for specific land-use/cover categories showed that pasture was increased with population density at the expense of forest cover (Fig. S8). Trends in pasture and forest use/cover changes were also apparent with varying market influence settings, which was a dynamic consistent with a land-use system in which pasture was the dominant land-use. Model runs showed a moderate level of variability in land-use outcomes within and across experimental settings, but variation increased markedly as population density increased and market influence decreased (Fig. S8). Finally, the genetic algorithm failed to find any parameter combinations that satisfied all three target behavioral patterns simultaneously.

S2.5. Site 1, Luoang Namtha, Laos

This site was a combined rice and swidden cultivation system in the Luoang Namtha region of northern Laos. Global environmental, demographic, and market data was re-sampled using the WGS 1984 UTM Zone 48N projection. Classified LandSat images from Heinimann et al. [4] were used as input land cover layers.

*S2.5.1. Results*

The model produced aggregate land-use/cover category counts and rankings that were roughly similar to those observed in the real landscape (Fig. S9). Generally, the lower the population density represented, the smaller the difference and more similar the rankings between real and modeled land-use/cover counts, and all experimental market influence-population density combinations performed better than the null model (Fig. S9). Disaggregating land-use/cover counts to land suitability classes demonstrated superior model performance compared to the null model for land suitability classes one through three (Fig. S9). Modeled distributions of land-use/cover categories were more similar to the real landscape than those of the null landscape (Fig. S10), and most accurate when close to the actual population density and market influence values of the real landscape. Variability in land-use outcomes ranged from zero to an almost 1,500 landscape cell difference (15 percent of landscape area) within a particular experimental settings. The trend was quite stark, as variability in model runs was zero until market influence was increased to 0.2 above the mean of the real landscape, at which point variability also increased with population density. The trend in genetic algorithm success was similar in that fewer successful solutions were found as market influence increased, but this occurred with decreasing population density.

S2.6. Site 2, Luoang Namtha, Laos

This site was located in a mainly swidden cultivation system in the Luoang Namtha region of northern Laos. The upper left corner of the landscape was located at 21°10'8.173'' N and 102°12'37.110'' E. Global environmental, demographic, and market data was re-sampled using the WGS 1984 UTM Zone 48N projection. Classified LandSat images from Heinimann et al. [4] were used as input land cover layers.

*S2.6.1 Results*

Similar to the other swidden site in Luong Namtha, Laos, the model produced land-use/cover category counts and rankings closely resembling those in the real landscape (Figs. S11). Rankings of land-use/cover categories were positively correlated with those of the real landscape across all experimental settings and performed better than the null model. Model performance improved across all experimental settings as population density increased, regardless of market influence levels. The same trend was apparent in the SEI of modeled land-use/cover distributions (Fig. S12). However, disaggregated distance measures based on land suitability class showed the opposite trend, as differences between real and modeled land-use/cover counts to land suitability area ratios were smallest with lower population densities (Fig. S11). While land-use outcomes within a given population density setting were not affected by changing market influence settings, the underlying variability in model runs across experimental settings increased markedly with increasing market influence (Fig. S12). Similar to the first Laotian site, variability was amplified by increasing population density. Finally, all 60 parameter sets generated by the genetic algorithm, across all experimental settings, satisfied all three target behavioral patterns simultaneously.

**References**

1. Ellis EC, Neerchal N, Peng K, Xiao HS, Wang H, Zhuang Y, Li SC, Wu JX, Jiao JG, Ouyang H, Cheng X, and Yang LZ (2009) Estimating long-term changes in China's village landscapes. Ecosys 12, 279-297.
2. Buck JL (1937) Land Utilitzation in China. The Unversity of Chicago Press.
3. Multi-Resolution Land Characteristics (MRLC) Consortium (2001). *2001 National Land Cover Data*. U.S. Environmental Protection Agency. Available at: http://www.epa.gov/mrlc/nlcd-2001.html.
4. Heinimann A, Messerli P, Schmidt-Vogt D, and Wiesmann U (2007) The dynamics of secondary forest landscapes in the lower Mekong basin: A regional-scale analysis. Mt Res Dev 27 (3), 232–241.
5. Boserup E (1965) The conditions of agricultural growth: the economics of agrarian change under population pressure. Aldine, Chicago: 124 pp.
